# Influence of water contamination on the sputtering of silicon with low-energy argon ions investigated by molecular dynamics simulations

**DOI:** 10.3762/bjnano.13.86

**Published:** 2022-09-21

**Authors:** Grégoire R N Defoort-Levkov, Alan Bahm, Patrick Philipp

**Affiliations:** 1 Advanced Instrumentation for Nano-Analytics (AINA), Materials Research and Technology Department (MRT), Luxembourg Institute of Science and Technology (LIST), 4422 Belvaux, Luxembourg; 2 University of Luxembourg, 4365 Esch-sur-Alzette, Luxembourg; 3 Thermo Fisher Scientific, Hillsboro, OR, 97124, USA

**Keywords:** angle dependency, argon ions, contamination, focused ion beams, ion bombardment, low energy, molecular dynamics, silicon, simulations, water

## Abstract

Focused ion beams (FIB) are a common tool in nanotechnology for surface analysis, sample preparation for electron microscopy and atom probe tomography, surface patterning, nanolithography, nanomachining, and nanoprinting. For many of these applications, a precise control of ion-beam-induced processes is essential. The effect of contaminations on these processes has not been thoroughly explored but can often be substantial, especially for ultralow impact energies in the sub-keV range. In this paper we investigate by molecular dynamics (MD) simulations how one of the most commonly found residual contaminations in vacuum chambers (i.e., water adsorbed on a silicon surface) influences sputtering by 100 eV argon ions. The incidence angle was changed from normal incidence to close to grazing incidence. For the simulation conditions used in this work, the adsorption of water favours the formation of defects in silicon by mixing hydrogen and oxygen atoms into the substrate. The sputtering yield of silicon is not significantly changed by the contamination, but the fraction of hydrogen and oxygen atoms that is sputtered largely depends on the incidence angle. This fraction is the largest for incidence angles between 70 and 80° defined with respect to the sample surface. Overall, it changes from 25% to 65%.

## Introduction

Focused ion beams (FIB) play an increasingly important role in materials research areas such as nanoanalysis (e.g., secondary ion mass spectrometry (SIMS) [1–3] and sample preparation for transmission electron microscopy (TEM) [4], atom probe tomography (APT) [5], and ion beam analysis used for life sciences applications [6–7]), surface patterning [8], nanolithography [9], nanomachining [10–11], and nanoprinting at room [12] and cryogenic temperatures [13]. The development of nanotechnology relies on lower ion beam energies and smaller spot sizes to reduce the thickness of the layer damaged by ion beams, and to increase the lateral resolution for precise machining and sample characterization. For most of these applications, the quality of the sample surface and its cleanliness are essential and, therefore, highly controlled. Depending on the application, the ion beam energy is in the range of 10 to 30 keV when small spot sizes are required (i.e., spot sizes in the nanometre range) and at a few keV or even in the sub-keV range when low surface damage or minimized atomic mixing is required. One example is low-energy depth profiling SIMS to resolve thin films in multilayered samples [14].

Another example is TEM sample preparation, where the achievement of the highest lateral resolutions in the subsequent TEM analysis requires lamellae thicknesses between 10 and 20 nm, but goes along with a typical amorphous layer of 2 to 4 nm formed during the sample preparation by FIB milling [15–17]. Such an amorphous layer represents a substantial part of the thickness of the sample and information coming from this part does not correspond to the initial sample structure. Minimizing the thickness of this amorphous layer during FIB milling is essential because most samples analysed in high-precision instruments are prepared using this method. This can be best achieved using low-beam energies, ideally in the sub-keV range [18], since low-energy ion beams (under 500 eV) produce a thinner amorphous layer due to their lower penetration depth. Investigations performed with low-energy argon ions [15,19–20] have shown that the current model describing the sputter yields and the sputtering processes (such as sputtering threshold and the amorphization process) does not fit with experimental data, leading to discrepancies that cannot be ignored.

Contaminants on the sample surface can also play a critical role during the milling process: water can be found on nearly every sample surface [21]. For example, in a vacuum chamber at a pressure of 10^−8^ mbar, there are still 10^6^ to 10^9^ molecules per cm^3^. This contaminant has a strong impact on the ion beam process by modifying sputtering processes. Even under an ultrahigh vacuum of 10^−12^ mbar there are still 10^4^ molecules·cm^−3^ remaining in the experimental chamber, thus making water by far the most common contaminant. These assumptions can be confirmed by SIMS experiments [22]. It is therefore safe to assume that most experimental chambers able to hold vacuum between 10^−6^ to 10^−10^ mbar will contain water molecules that will interact with the sample surface. Water has been widely studied [23–26] and is well described by many simulation tools available to model samples in such extreme cases.

Nowadays, very little is known about the influence of contaminations on the amorphization process under ion irradiation. Thanks to molecular dynamics (MD) simulations, a wide range of materials properties and process parameters can be reproduced [27–29]. In this paper, we are using MD simulations to study the sputtering of a surface with water contamination by sub-500 eV ions. The information of interest is the chemical reactions occurring at and below the sample surface, as well as the mixing of the contaminant layer into the sample. The ReaxFF reactive force field is used in this work [30]. ReaxFF force fields are specifically tuned for a set of atomic interactions. They are developed from quantum calculations and are adapted for MD simulations, providing faster calculations than pure quantum electrodynamics (QED)/density functional theory (DFT) and more information than classical MD/binary collision approximation (BCA) simulations. ReaxFF simulations can cover a broad range of applications: from DNA molecule bombardments with heavy atoms [31] to graphene layer deposition on copper surfaces [32]. Numerous simulations using the ReaxFF potential have been conducted in the past years, several describing a given sample with contaminant interactions (such as water or organic particles) [33–35].

To understand how the water layer influences the amorphization process, we designed metrics aiming to characterize the degree of amorphization in the sample. Previous works have tried to give a broader picture of the phenomenon in solids or in crystals [36], by using a very small sample size and performing an exhaustive study on bond orders and bond length average (Gaussian core model), as well as analysing the granularity of the system in liquid/solid phases. However, these methods were designed using a limited sample size of up to a few hundreds of atoms. Other studies have tried to elaborate a local metric for condensed-phase environments [37], giving a good descriptor for phase transitions. While they provide good results for a specific crystalline structure, they are tedious to adapt to other structures. In this paper, we developed a simple metric based on bond length variations, which can be easily adapted to different crystalline structures to characterize sample amorphization and also to study sputtering surfaces exposed to residual water molecules.

## Computational Methods

### Force fields

The ReaxFF force field differs from other established force fields as it aims to bridge quantum mechanics (QM) and classical MD. Quantum mechanics algorithms are limited to small-sized samples (up to a few hundreds of atoms) due to the difficulty to solve Schrödinger equations for many-body systems whereas classical MD makes the assumption of fixed bonds and usually do not include complex chemical reactions. ReaxFF captures the dynamic nature of bond formation and breaking, based on bond-order calculation from interatomic distances. ReaxFF also includes nonbonded terms such as van der Waals and Coulombic interactions in the energy calculation, resulting in a sum of partial energies [38]. The ReaxFF force field was developed by van Duin et al. [34] for the Si–C–O–H system, and for all simulations described in this paper, ReaxFF_SiOCH(2019)_ is used. To reproduce the interaction between argon ions and silicon, hydrogen, and oxygen atoms, we used DFT to simulate the potential energy between each of these pairs: Ar–Ar, Ar–Si, Ar–H, and Ar–O. Once extracted, the potential energy was fitted using the Morse potential which is described in Equation 1:


[1] $$E_{\text{syst}}=D_{0}\left[e^{-2\alpha \left(r-r_{0}\right)}-2e^{-\alpha \left(r-r_{0}\right)}\right],$$

where *r* represents the interatomic distance in distance units, *r*_0_ the interatomic distance in the equilibrium state (also in distance units), *D*_0_ the depth well in energy units (defined per atomic interaction and related to the molecule dissociation energy), and α the control parameter on the well thickness, proportional to the curvature of the potential at its origin, in inverted length units [39].

The DFT data has been obtained using the VASP [40–43] package. The Perdew–Burke–Ernzerhof generalized gradient approximation (GGA-PPE) [44] is selected together with the projector augmented wave (PAW) [45–46] method for the pseudopotentials describing core electrons of the different elements. Van der Waals contributions are included using the Tkatchenko–Scheffler method [47]. A plane wave cut-off of 500 eV is used and k-points are automatically generated. The self-consistent calculation is performed using a Davidson block iteration scheme with a threshold of 10^−6^ eV/atom. The force threshold for the conjugate gradient geometry optimisation is set to 10^−4^ eV/Å. The interatomic interactions are calculated by changing the distance between Ar and H and between O and Si atoms by 0.1–12.0 Å in 0.1 Å steps in a simulation box of 25 × 10 × 10 Å^3^ by taking the energy for a couple of Ar–X interactions by the energy of the two atoms taken individually.

**Figure 1:**
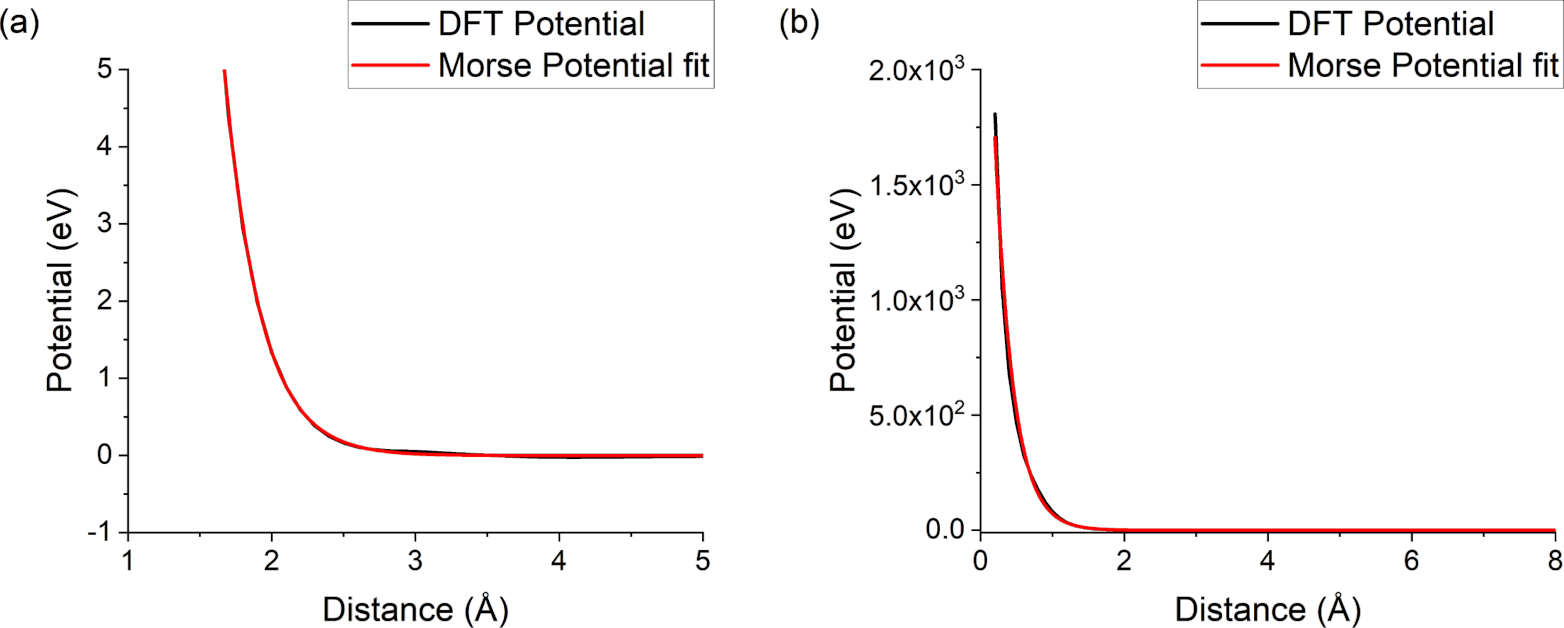
Fit of the DFT data for the argon–silicon potential in (a) the 1–5 Å region, with the potential well, and (b) the entire potential.

The fit of the Morse potential can be seen in Figure 1 for the Ar–Si bond, and the remaining data of the fits can be found in Supporting Information File 1. The Ar–X interactions have a potential well in the order of a few meV, which makes it rather difficult for the fit to precisely match the well. However, this is not problematic as the potential well is in the order of magnitude of thermal vibrations and its impact on the outcome of the simulations is small. The fits match the repulsive part of the potential for the Ar–X couples well, except for high energies. However, for energies up to 100 eV discrepancies are small so that the interpolation to the ZBL potential at short interatomic distances can be omitted*.*

The ReaxFF potential is used to model the damage cascade and the collisions between silicon, hydrogen, and oxygen particles. An hybrid pair style [48] was used in the MD simulations to match both the ReaxFF potential (for Si–Si, Si–O, Si–H, O–O, O–H, and H–H bonds) and the Morse potential (for Ar–Si, Ar–O, Ar–H, and Ar–Ar interactions). The charge equilibration for the ReaxFF potential was performed using the algorithms developed by Rappé et al. [49–51], namely QEq, allowing charge distributions to be calculated according to geometry. Due to the dynamic nature of bonds and geometrical configurations, the charge distribution QEq is performed every 10 time steps during the simulation of ion bombardment, allowing to cut down computation costs while maintaining a good fidelity to the charge distribution. Furthermore, we implemented a variable time step, which allowed us to control the variation in either position (with a limit from 0.01 to 0.1 Å) or energy (up to 0.4 eV) in which the time step would be reduced in order to take these variations into account. As a result, a maximal displacement of one interatomic distance observed in the simulation could not occur in a time frame smaller than approximately 40 time steps, which reassured us regarding the 10 time step limit for QEq. During water deposition on top of the silicon sample, the charge equilibration was performed at each step to fully describe the deposition of the contaminant on top of the sample.

### Molecular dynamics simulations

**Figure 2:**
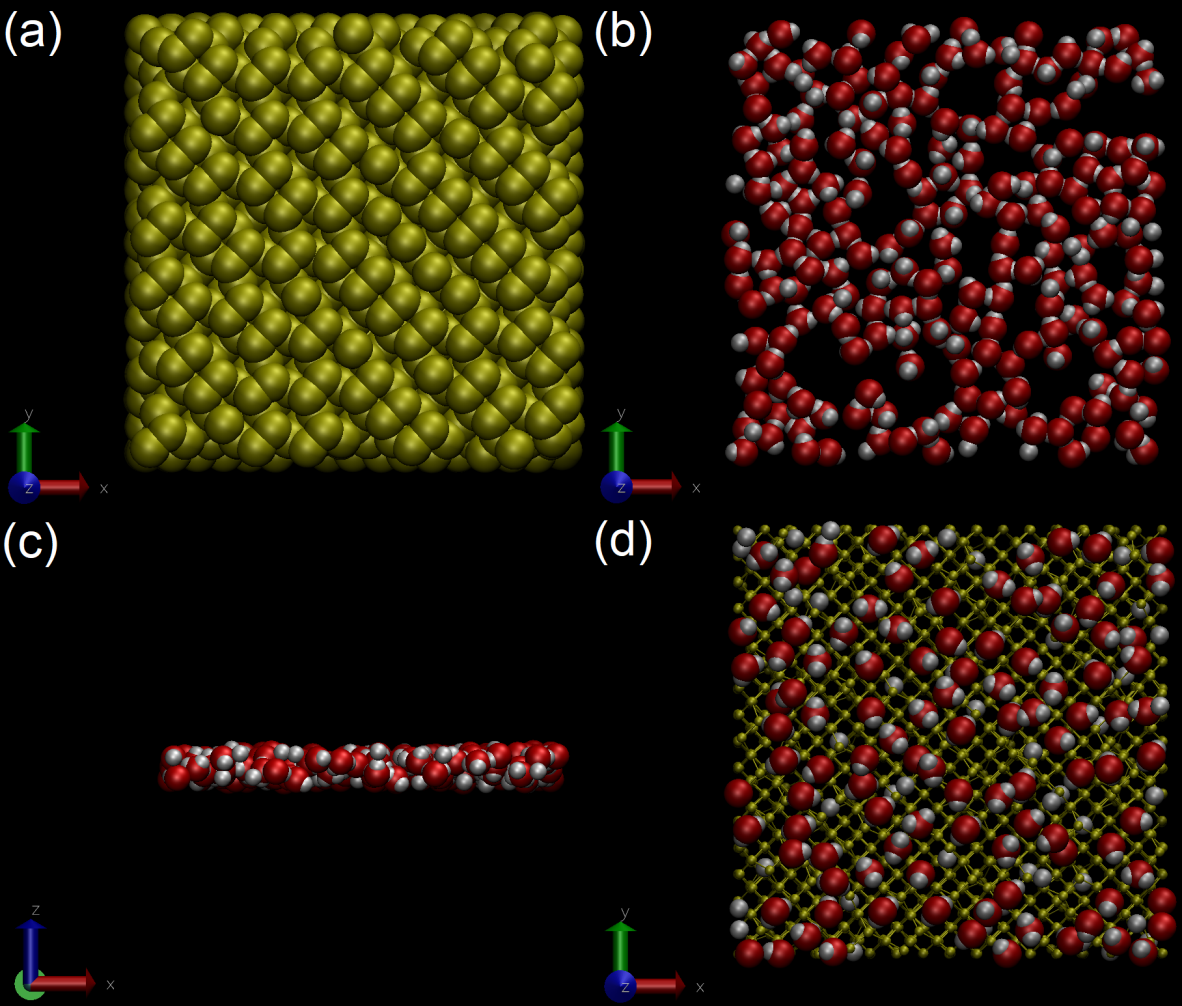
Visualisations of the samples during equilibration steps: (a) represents the silicon sample after Stillinger–Weber equilibration, showing dimers on the surface with a VDW representation; (b) represents the water layer after equilibration (top view), while (c) shows the side view, both in a VDW representation. (d) Final sample once equilibrated with both the water layer and the silicon sample using ReaxFF and energy minimized with water molecules in a VDW representation, and silicon atoms in a CPK representation. The direction of the *z* axis is defined by the blue arrow, the *y* axis by the green arrow and the *x* axis by the red arrow.

Molecular dynamics simulations of argon bombardment on a silicon surface were carried using the LAMMPS code [52]. A pristine silicon sample containing 5248 atoms was created and used as a reference sample. On top of this sample was added a water layer to study the effect of contaminations. Periodic boundary conditions were applied in *x*- and *y-*directions, with some vacuum above and below the sample in the *z*-direction. The different axes are defined in Figure 2.

The simulations for sample preparation were run in an NVT ensemble using the Langevin thermostat with a damping parameter set to 100 time steps and the temperature set to 300 K [53]. The pristine sample was equilibrated using the Stillinger–Weber potential to obtain the dimers formed on the Si(100) surface [21,54], with a time step of 0.1 fs for 1 ns. Some snapshots of the dimers on the surface can be found in the Figure 2.

The equilibration was pursued with the ReaxFF potential for another 500 ps in the same aforementioned conditions. The largest displacements observed in the sample were vibrational motions due to a temperature of 300 K (between 0.5 to 1 fs), and the time step was chosen to limit the maximum displacement between steps to one tenth of that motion, resulting in a 0.1 fs time step for the simulations. Furthermore, as ReaxFF tends to reach a better stability at a shorter time step, a variable time step was defined between 0.05 and 0.1 fs, with a maximal allowed energy variation per atom between each step of 5 eV. This process allowed to limit the maximum displacement of atoms per time step while the damage cascade happened and to increase it after the energy dissipated into the system. During the ion irradiation simulations, the bottom slab of the sample (one lattice high) was set in an NVT ensemble using the same methodology in order to reproduce a heat-bath effect [55]. The remaining atoms were kept in the NVE ensemble in order to avoid any impact of a thermostat on the collision cascade.

**Figure 3:**
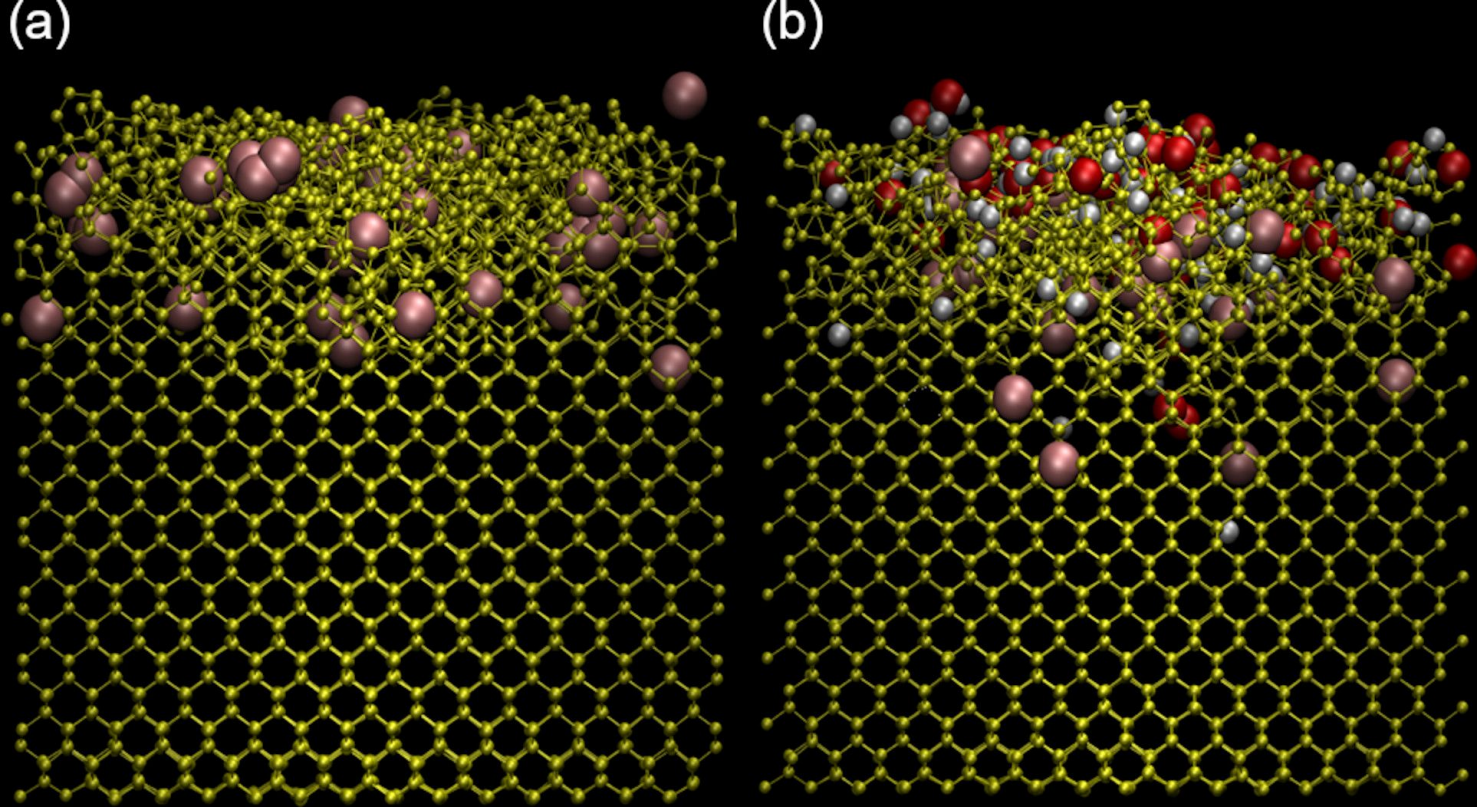
Representations of (a) clean and (b) contaminated samples. The silicon atoms are shown in yellow, oxygen atoms in red, hydrogen atoms in white, and argon atoms in pink. Both samples were bombarded at 45° and 100 eV and are viewed from the (110) direction after 500 irradiation events.

Once properly equilibrated, the simulation of argon bombardment of pristine Si(100) was run for 50 ps, which was enough for the modelling of energy dissipation and sputtering, and then thermalized to 300 K. The last script was set to end as soon as the computed mean temperature reached 300 K. The whole process was repeated until 500 ion impacts had been simulated. Compared to experiments, the time between two ion impacts needed to be reduced by several orders of magnitude due to computational constraints (i.e., a few hundred picoseconds in simulations compared to µs in experiments) leading to an overall higher irradiation density yet keeping the sample at room temperature. To reproduce an ion beam, the initial positions of the argon ions were randomized in the surface plane. Each simulation set consisted of 500 irradiation events at a set incidence angle and at an impact energy of 100 eV. For a surface area of 18.9 nm^2^, this corresponds to a fluence of 2.6 × 10^15^ atoms·cm^−2^. The simulations were carried out for incidence angles of 0, 20, 30, 45, 65, 72, 75, 83, and 85° with respect to the surface normal. These angles were selected to cover a good sample in the 0–85° range, including angles of specific interest, such as 72° and 83° which represent a channelling angle for Si(100) [56] and a grazing incidence angle, respectively, frequently used in TEM lamellae preparation [57]. A visualization of a set of simulations can be seen in Figure 3 and the global parameters of the simulations can be seen in the Table 1. Typical values for fluence in experiments are between 10^15^ and 10^17^ ions/cm^2^.

**Table 1:** References of the simulation parameters.

	Surface (nm^2^)	No. of impacts	Energy (eV)	Fluence (ions/cm^2^)

simulations	18.9	500	100	2.6 × 10^15^

To prepare a contaminated sample, a slab of water was equilibrated first and then deposited onto the Si sample. The water molecules were equilibrated using the ReaxFF_OH(2017)_ [24] potential in a fully periodic space using the NPT ensemble. Since the adsorption of water molecules on a surface is a random process, the orientation of the molecules could freely evolve. Snapshots of water equilibration can be found in Figure 2. Next, the potential was switched to the ReaxFF SiOCH potential [34] used for the bombardment simulations, followed by another energy minimization. The change to ReaxFF_SiOCH(2019)_ did not induce any significant change during the potential change, the main visible one being a slight variation of the bond length O–H in the water molecules that did not directly interact with silicon particles (i.e., water molecules on top of the contaminant layer). Thereafter, the water layer was deposited on top of the pristine silicon sample in the NVE ensemble for up to 1 μs in order to reproduce the gradual contamination of the sample by residual gas molecules, by giving a vertical energy of 0.5 eV to the water slab. After water deposition, a series of irradiation simulations was carried out using the same conditions than those for the pristine sample.

For the estimation of water contamination, we based our simulations on instrument chambers with a vacuum level between 10^−8^ and 10^−6^ mbar and a sample area of 18.9 nm^2^. According to [58–59] water forms about 10% of the residual gas in the experimental chamber. By using the Hertz–Knudsen formula (Hertz–Knudsen formula, p. 159 in [60]) we can determine, for a specific pressure, the temperature and atomic mass of a molecule and the number of collisions per second and per square centimetre:


[2] $$Z_{a}=3.51\times 10^{22}\left(\frac{P}{{\left(T\cdot m\right)}^{0.5}}\right),$$

where *Z_a_* is the number of collisions per second, *P* is the pressure in Torr, *T* is the temperature in Kelvin, and *m* is the atomic mass of the molecule of interest in amu. With *P* = 7.5 × 10^−8^ Torr, *T* = 300 K, and *m* = 18.02 amu we obtain a value for the collisions per square centimetre of *Z_a_* = 3.58 × 10^13^ collisions·s^−1^·cm^−2^. Once scaled to the sample size, we obtain *Z_a_* = 6.61 collisions·s^−1^·surface^−1^, from which about 10% would be water collisions. Hence, for typical vacuum conditions a monolayer of water is formed within minutes.

## Results and Discussion

### Sample characteristics and contaminant effects

**Figure 4:**
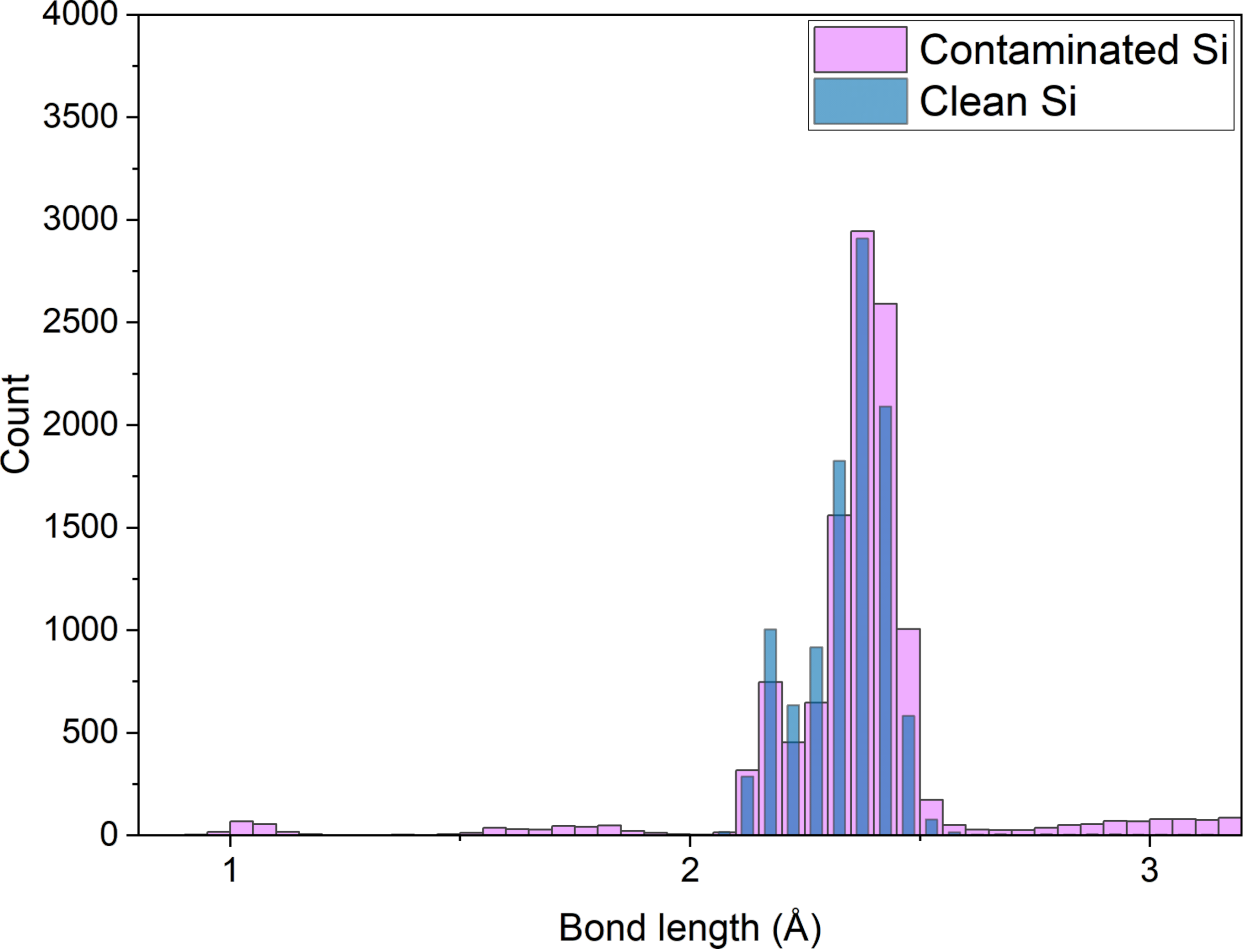
Bond length distributions for the pristine sample (blue) and the contaminated sample (pink) regardless of the particle type with a cut-off at 3.2 Å (at which distance the distribution for the 2nd neighbours starts).

At first, bond length distributions were calculated to observe the effect of the water contaminant on these distributions. The nominal Si–Si bond in a diamond lattice is 2.358 Å. Figure 4 shows the distributions for both samples before ion bombardment. For the Si–Si bonds, the most probable bond length is at the correct length, but a significant number of bonds are in the 2.1 Å region. This can be explained by dimers appearing on top and bottom surfaces which have a nominal bond length (for Si dimers) of 2.1 Å.

**Figure 5:**
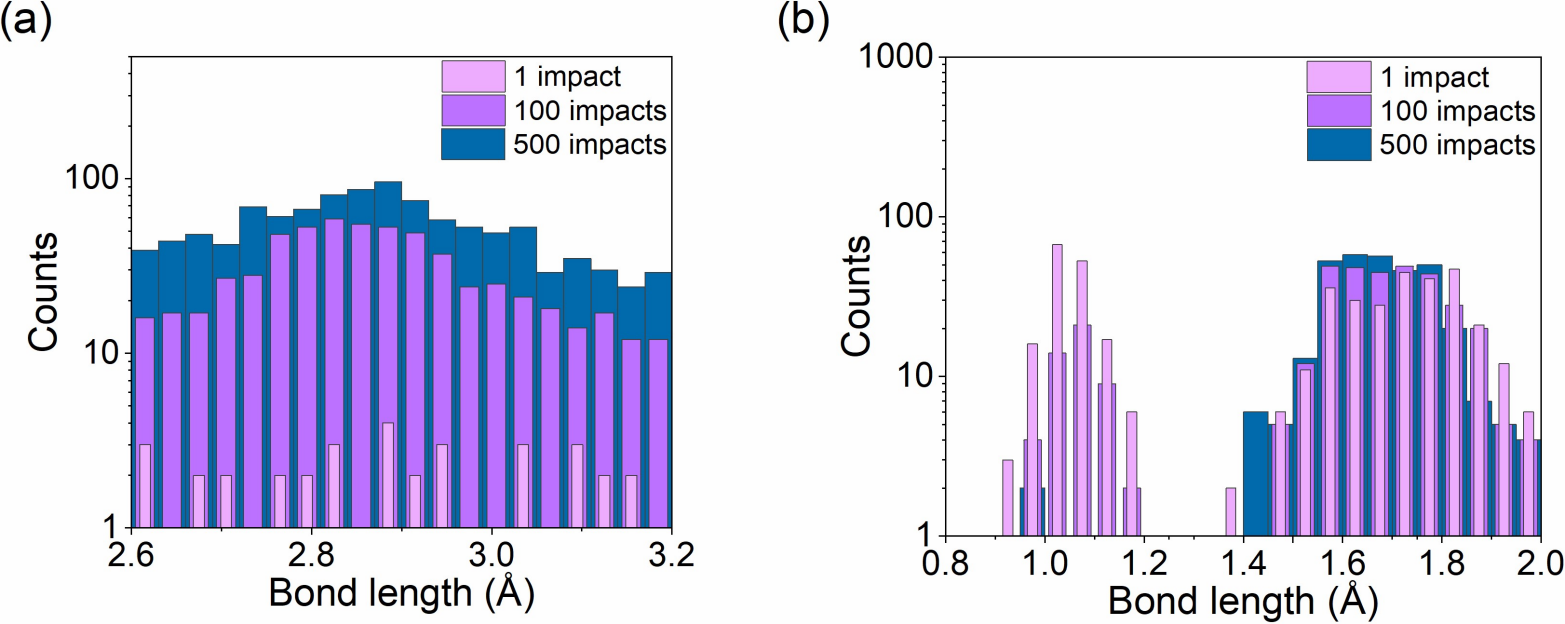
Evolution of bond lengths in the contaminated sample under bombardment. (a) The first graph shows bonds between 2.6 to 3.2 Å describing the amorphous layer formed by Si–Si bonds, and (b) the second graph shows bonds between 1 and 2 Å describing O–X and H–X bonds.

The distribution for the contaminated sample shows two additional regions: the region between 0 and 2 Å and the region between 2.6 and 3.3 Å. The smallest bond length around 1 Å is due to O–H bonds [61] in water molecules. The second peak observed at approx. 1.4 to 1.7 Å is associated to both Si–H (1.56 Å) [62] and Si–O (1.58 Å) [63] bonds. The Si–H bonds are located near the surface between Si dimers and some of the H atoms of water after the reaction between silicon and oxygen, leading to the splitting of water molecules. Studies have suggested that oxygen dissociates on a single Si–Si dimer, allowing for the recombination of dissociated hydrogens with Si surface atoms [64]. Some counts are also measured between 2.6 and 3.2 Å, which come from the amorphization of regular Si–Si bonds due to argon collisions. Since most of the Si–O and Si–H bonds are shorter than these counts, it is safe to assume that the local disorder induced by reactions between water molecules and the sample surface tends to disorganize the silicon lattices. These bonds are longer than those of the crystalline structure, and their distribution follows a Gaussian distribution. Both distributions are depicted in Figure 5, showing the evolution of the counts with respect to the number of bombardments (i.e., with increasing fluence).

Furthermore, Figure 5 shows how 500 impacts modified bond length distributions: the counts related to amorphous bonds (between 2.6 and 3.2 Å) have clearly augmented, with a reduction in the O–H bond counts (for the contaminated sample). Hence, most of the water adsorbed on the sample surface is either fractioned and mixed in the sample with repeated argon atom impacts, creating more Si–H and Si–O bonds, or sputtered from the sample surface.

### Description of the amorphization

**Figure 6:**
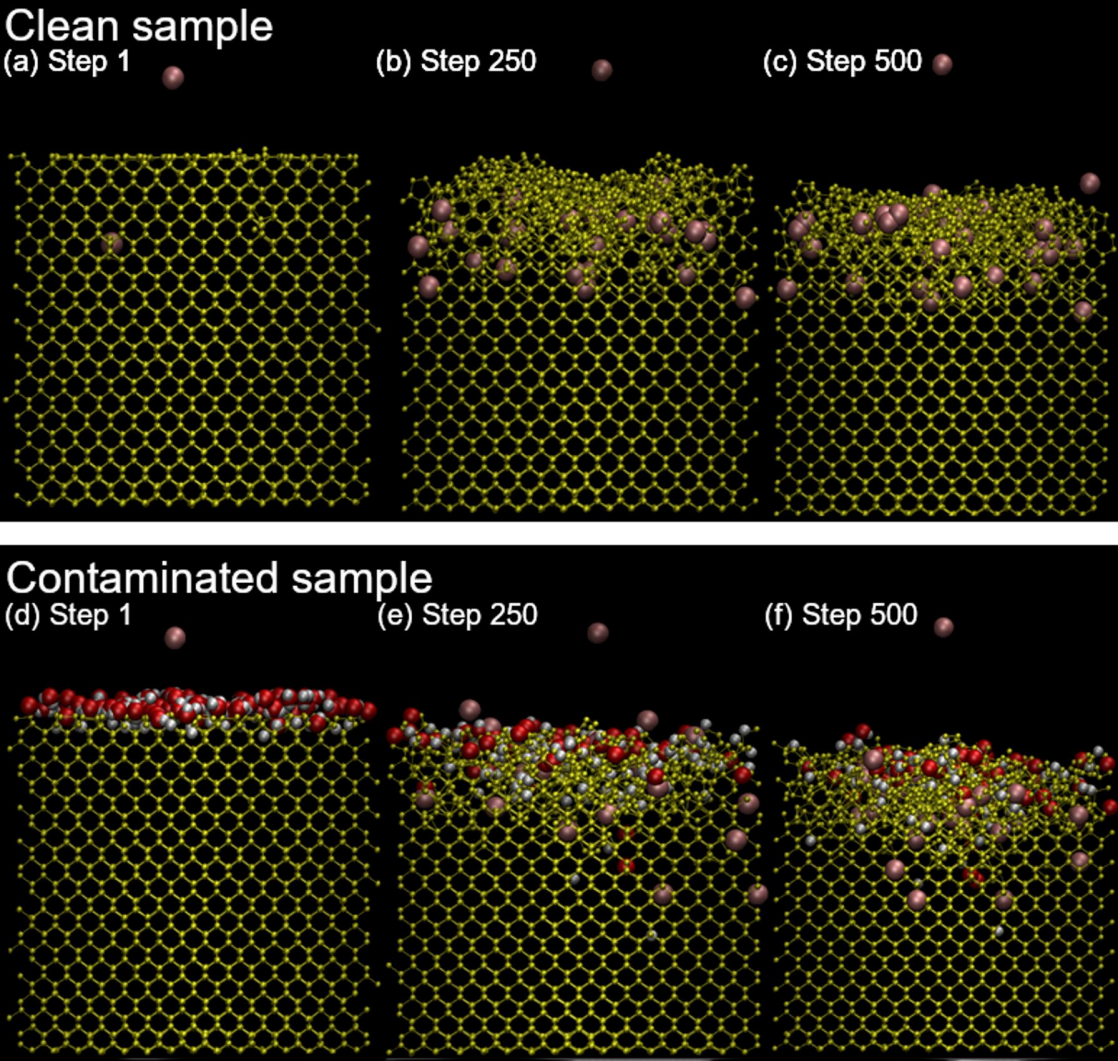
Evolution of the clean (a, b, c) and contaminated (d, e, f) samples under ion irradiation, before the 1st, 250th, and 500th bombardment showing the evolution of the sample surface. The argon particle on top of the simulation is the ion which is bombarded onto the sample.

The two samples at several stages of the sputtering process are shown in Figure 6 to illustrate the evolution of the amorphization process. The penetration depth of argon atoms is limited to the first few lattices inside the silicon, for both the contaminated and clean samples. For the clean sample, the amorphization depth linearly evolves until the implanted argon atoms reach a saturation concentration due to the diffusion and desorption of excess atoms. In the contaminated sample, the observations are similar for argon and for the contaminants, and hydrogen is implanted deeper than oxygen. Both atoms also tend to be implanted deeper than argon, proportionally to their respective masses, but some occurrences of deep argon implantation can happen. The amorphization can be divided into two phases: in an initial phase, most silicon atoms stay in their lattice position, even after receiving some energy from the incident argon ion, and only a small number of defects is formed. Water molecules are usually fragmented by an argon ion on the sample surface. The hydrogen and oxygen atoms are lighter than argon and are pushed deeper into the sample, globally increasing the damage per collision. The argon ion is either backscattered or implanted. Previously implanted argon atoms can also diffuse to the sample surface when receiving enough energy. The different processes gradually move deeper into the sample with increasing fluence. The second phase is characterized by several impacts which happen in close surroundings of a given region, and most atoms are displaced from their initial equilibrium position. This phase describes an amorphous layer. The depth of the amorphous layer varies as a function of the incidence angle and is maximum at around 70° with respect to the surface normal. In the amorphous region, contaminants and argon atoms reach a saturation concentration. Once the argon saturation is reached, the thickness of the amorphous layer reaches a maximum, which is defined by the range of the argon atoms. At the same time, material is removed by sputtering from the surface.

### Amorphization coefficient

To properly describe the amorphization process, we choose to monitor how the bond length distribution changes with the number of impacts. In particular, we calculated the coefficient described in Equation 3:


[3] $$\mu =\frac{1}{n_{\text{Bonds}}}\sum_{n_{\text{Bonds}}}\left[1-\left|\frac{{\text{BondLength}}_{\text{Theory}}-{\text{BondLength}}_{\text{Measured}}}{{\text{BondLength}}_{\text{Theory}}}\right|\right].$$

To characterize the amorphization process as a function of depth, the simulation box was split into different slabs along the *z*-direction, each slab having the height of one-unit cell and the initial slab being situated at the bottom of the sample with an undisturbed surface. Then, the number of bonds and bond lengths in each slice are calculated to obtain the µ coefficient associated to each slab. By comparing the different values of µ to the radial distribution functions (RDFs) of the different slabs, the data can be grouped into three different categories: the crystalline slabs, the semi-amorphous slab(s), and the amorphous slabs (see discussion on RDF for more details). The 0.94 < µ < 1 interval describes a region where no defects are present. Changes in µ are due to variations in bond length coming from thermal vibrations around the equilibrium bond length. This region is crystalline. The 0.89 < µ < 0.94 interval describes regions that contain defects but are not completely amorphous (i.e., the crystalline structure is preserved at least in a small part of the slab). The collisions have only caused minor damages. For simplicity, we call this transitional phase a “partially amorphous” or “semi-amorphous” region in the discussion. The µ < 0.89 region designates slabs with almost no remaining crystalline structure. There is a large variance in bond lengths, generally describing a damaged/amorphous area with a lot of disorder.

**Figure 7:**
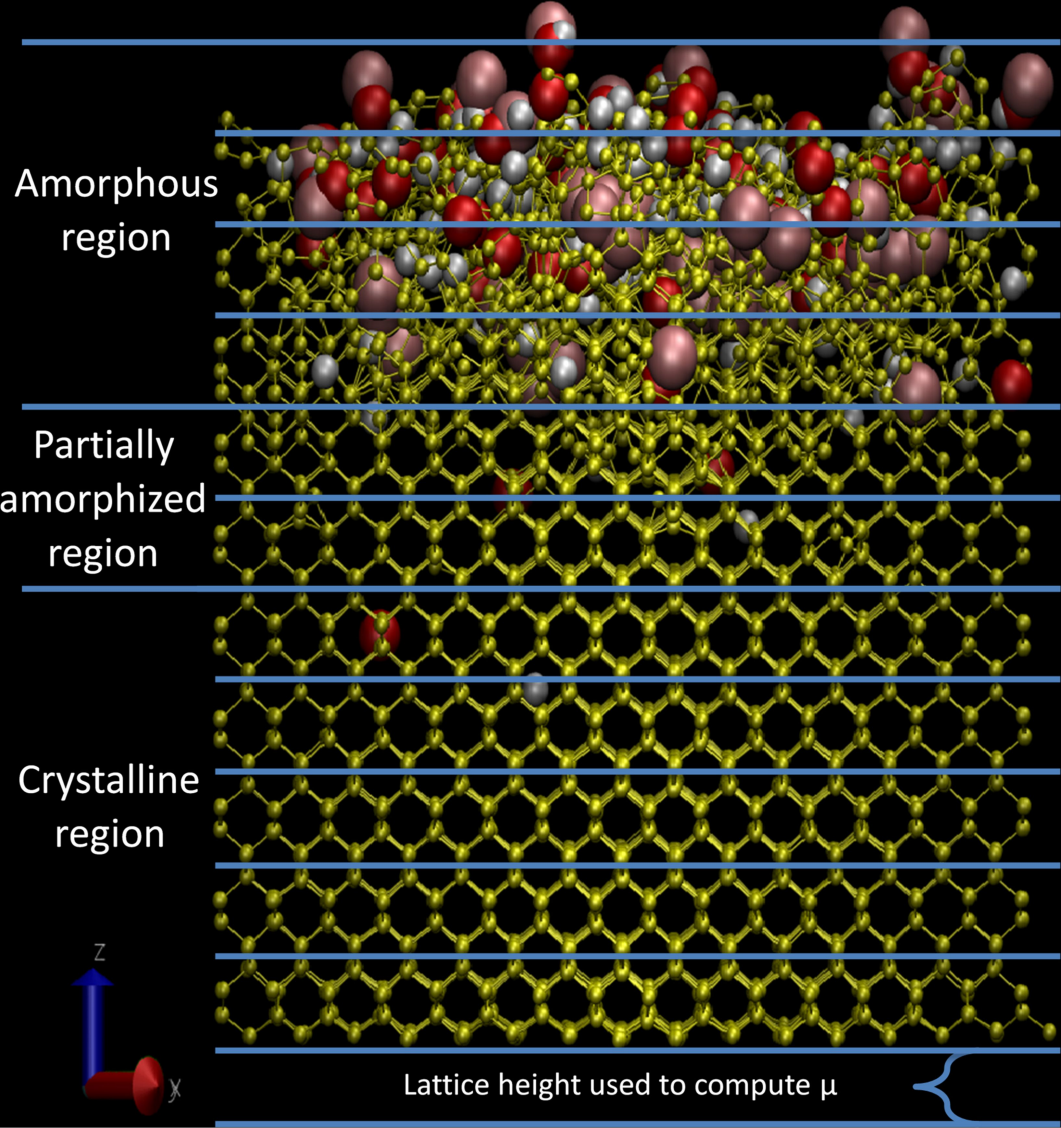
Representation of each region in the sample, the amorphous region is the one with the highest disorder, the crystalline region is the one with the lowest disorder, and the partially amorphized region represents the transition between the two regions.

**Figure 8:**
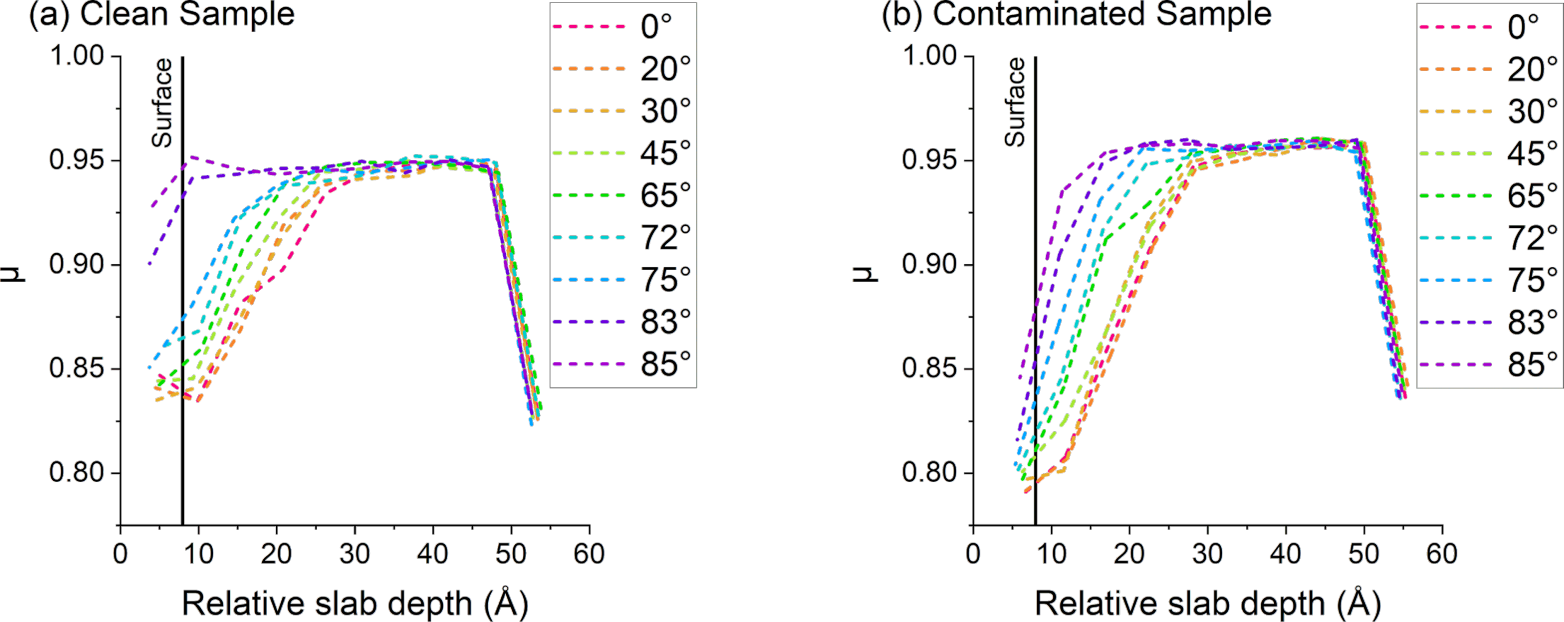
Evolution of the μ coefficient with respect to the angle for (a) pristine and (b) contaminated samples. The last data point at 55 Å has a low value in the 0.825–0.850 region due to the non-periodicity along the *z* axis in the simulation, causing the formation of dimers on the bottom of the sample.

A full breakdown of a sample bombarded by 0°, 45°, and 75° argon ions and the associated µ coefficients can be found in Supporting Information File 1, where each slab is detailed and compared with the associated RDFs. As per design, the “semi-amorphous” layer is supposed to be one-slab thick, as it is the transition between the amorphized region and the crystalline/intact part of the sample. In some cases, deeper penetration through the channelling of some atoms can cause a few slabs to be in the semi-amorphous regime. Figure 7 shows the three regions in the sample, and Figure 8 represents the evolution of µ in the sample with respect to the depth of the slab as well as with respect to the angle, describing how the incidence angle of the ion influences the amorphization depth. The sample shows a continuous transition from crystalline to amorphous. A large difference is observed between pristine and contaminated samples close to the grazing incidence. For the pristine sample the material is barely amorphized, whereas the presence of the contaminant leads to a full amorphization. In general, the incidence angle changes the depth at which the transition between amorphous and partially amorphized layer happens. Close to normal incidence the demarcation is clear. At larger angles, the transition is progressively shifted closer towards the surface. Especially for angles above 83° there is almost no amorphous layer, the upper part of the sample is at most partially amorphized by the collisions. This can be explained by the fact that at such high angles the argon particles are mainly backscattered and interact very weakly with the sample. In the case of the contaminated sample, most of the damage is done via the fractioning of water molecules on the sample surface. The implantation of argon ions is minimal.

Compared to other existing methods, our solution only uses the nearest neighbours of a given atom, which allows for a faster computation speed when using a large number of atoms. The results show that with increasing disorder the amorphization can be described by the change in bond lengths and bond angles. Therefore, the variation and distribution of angles in the amorphous region evolve to a point where all the angular distribution information becomes irrelevant. A methodology based on the averaged bond order parameters would hence become very time consuming for a larger-scale sample (compared to the methodology by Lechner and Dellago [36]). On the other hand, a method using a local-order metric as described by Martelli et al. [37] can be used for a whole sample. However, in the current study the information on the transition of the crystal structure between different regions cannot be obtained. For these reasons, we developed our methodology (i.e., a bond-centric analysis based on the definition of regions). Furthermore, by using a local-order metric*,* a high degree of overlapping would make it difficult to observe the precise region where the transition between each previously defined region would happen.

### Radial distribution function

**Figure 9:**
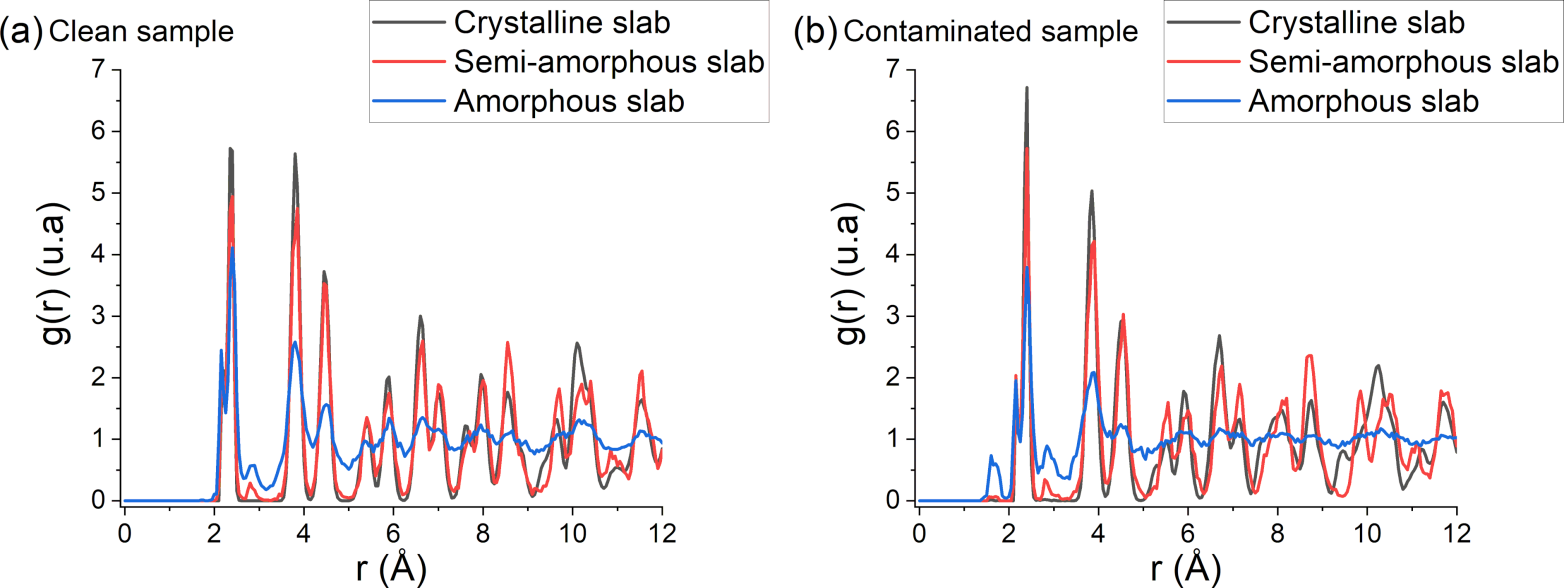
Radial distribution function for (a) clean and (b) contaminated samples for all previously determined regions. The limit fixed to 12 Å represents 2.5 lattices (or more than 10 neighbours).

The RDF has been calculated for different slabs to support the findings of the previous section. The algorithm used to calculate RDF in finite non-periodic samples can be found in the article written by Kopera and Retsch [65]. With the previously defined slabs, it is possible to calculate the RDF for each region, showing clear differences in the local structural coherency. Figure 9 shows the RDF for each slab in pristine and contaminated samples. A first observation is the difference between RDFs of each slab: the amorphous layer has almost no coherency whereas the crystalline slabs have a strong local order and patterns repeating past the 2nd nearest neighbours (i.e., for crystalline structures the peaks above 5 Å are still well defined while different peaks are not separated for the amorphous structure). The semi-amorphous region shows some coherency after the 2nd neighbour peak but with some characteristics of amorphous RDF.

**Figure 10:**
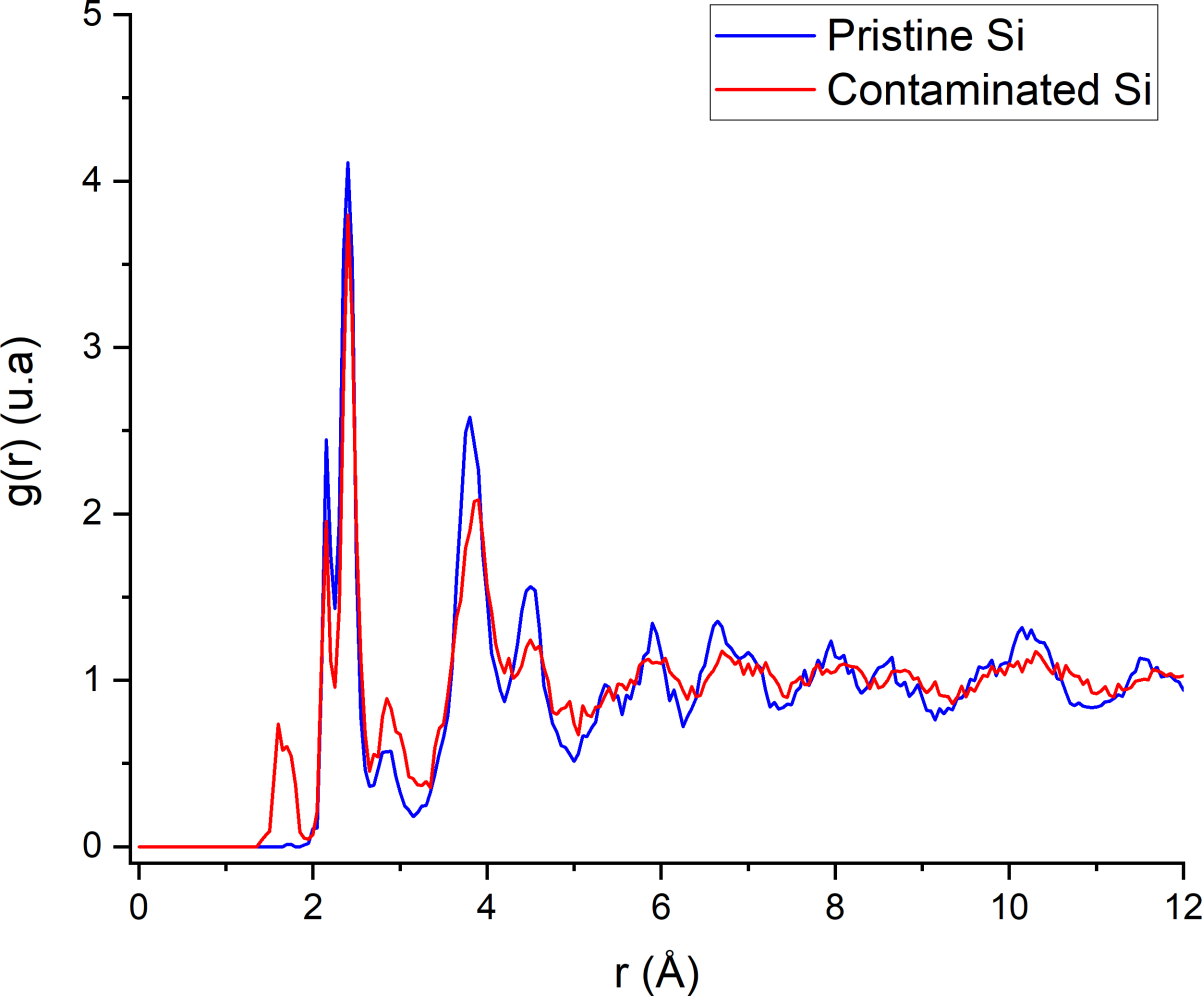
Comparison of the radial distribution function for both samples in the amorphous layer. The same cut-off of 12 Å is applied.

**Figure 11:**
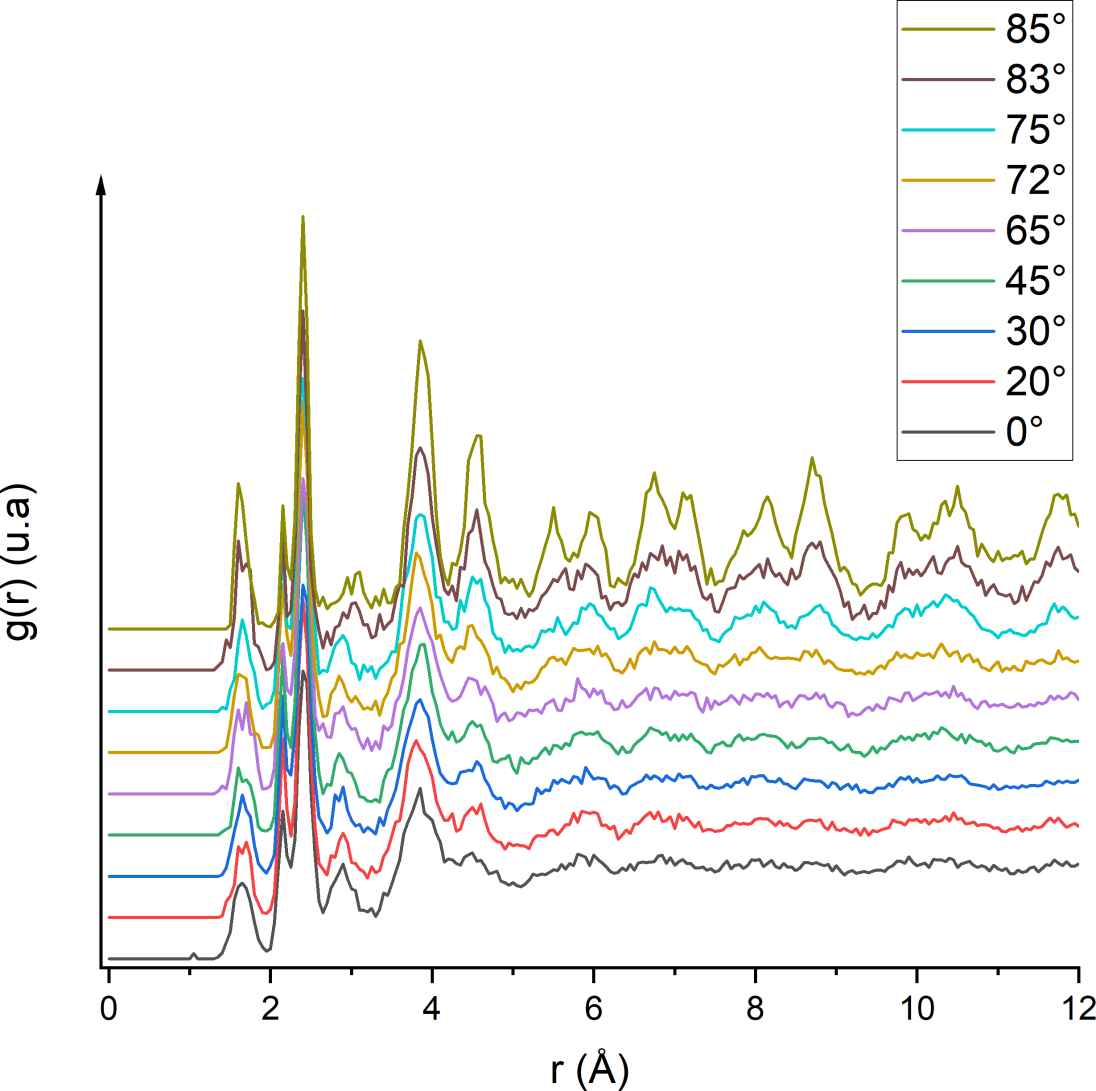
Comparison of the radial distribution function in the amorphous slab of the contaminated sample with respect to the angle of impact. The angles selected for this study are listed above and aim to cover values between 0° and 85°.

We can also observe the difference between clean and contaminated samples in Figure 10: For the clean sample, a smaller peak appears between the 2nd and 3rd peaks in the 2.6 to 3.2 Å region. These bonds are due to defect production in silicon after argon irradiation. For the contaminated sample, the same peak can be observed and another peak appears in the 1.5 Å region, which is created by the Si–H and Si–O bonds due to fragmentation of water molecules. From these observations we can determine the influence of the incidence angle on the amorphization process by comparing the RDF of a slab (Figure 11). When comparing the RDF at grazing angles for the clean and contaminated samples, there is less damage directly done by argon ions. However, more damage comes from hydrogen and oxygen atoms mixed into the sample. Due to grazing incidence, most argon ions are backscattered; however, the deposited energy is high enough to split the water molecules and push hydrogen and oxygen atoms deeper into the sample. As hydrogen is very light it can travel further into the sample, channel through the lattices, and preferentially go into interstitial sites. The same reasoning applies to oxygen except that it implants at a lower depth due to a higher mass which makes channelling less likely. The Si–O bonds are also stronger than the Si–H bonds, which leads to different implantation depths for both particles: oxygen particles are caught more easily by silicon atoms and, once formed, it requires a higher energy to break stronger Si–O bonds. Hence, this leads to a shallower implantation of oxygen into the sample. At higher angles, the RDF of an amorphous slab is almost identical to that of the semi-amorphous region, meaning that the disorder created by the collisions is marginal and the variations of the µ coefficient of the slab are minimal. In the next section, the implantation depths of argon, hydrogen, and oxygen are discussed regarding their impact on the amorphization process.

### Ion implantation

**Figure 12:**
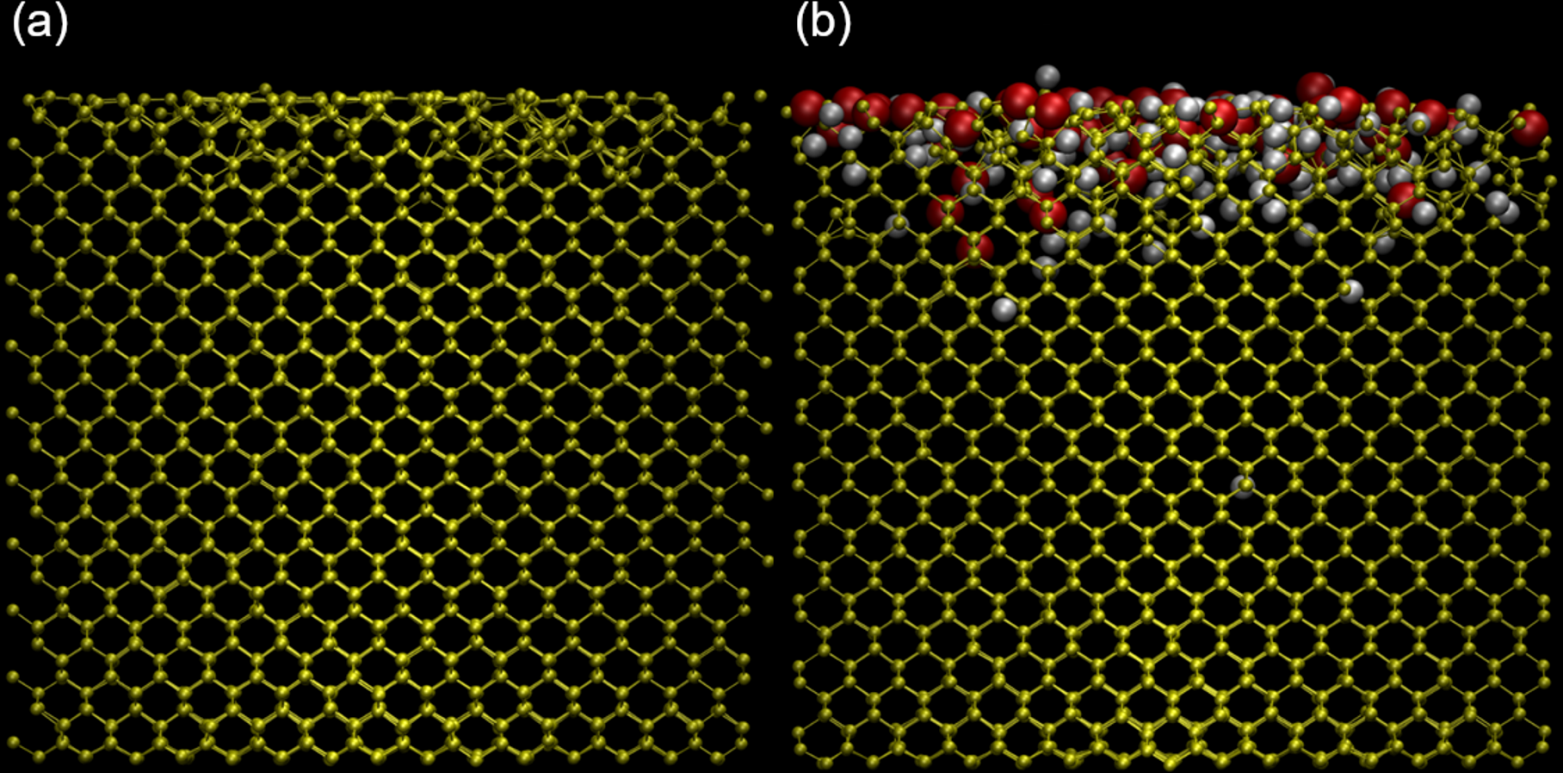
Comparison between samples after irradiation with an incident beam at 100 eV and 85° for (a) the pristine sample and (b) the contaminated sample, where the silicon atoms are in the compact representation (yellow). The oxygen (red) and hydrogen (white) atoms are represented with their van der Waals interaction sphere.

The implantation depth of each chemical element (e.g., hydrogen, oxygen, and argon) and the number of implanted atoms greatly vary with respect to the incidence angle. At grazing incidence, argon is not implanted but rather backscattered. For the contaminated sample, the argon can fragment water molecules even at grazing incidence. Hydrogen and oxygen atoms displaced at the same surface by an incident argon ion are either sputtered or implanted into the sample, causing significantly deeper damage than that observed for the pristine sample, as shown in Figure 12. At low fluences, most atoms are not involved in collision cascades. However, with increasing fluence (i.e., with the number of impacts going up to 500) almost all atoms of the surface will be displaced from their initial position and all water molecules will be eventually destroyed. After getting some energy in an atomic collision, the direction of hydrogen or oxygen atoms allows for channelling in some situations, leading to a deeper penetration into the bulk without causing damage.

The biggest difference between pristine and contaminated sample is also observed in these high angles (80° and above), where in the pristine sample most of the interactions are located on the surface, and in the contaminated sample hydrogen and oxygen are causing damage deeper in the sample. Since these angles are commonly used for the final polishing in TEM lamellae preparation, it is interesting to understand the impact of water in this specific case.

**Figure 13:**
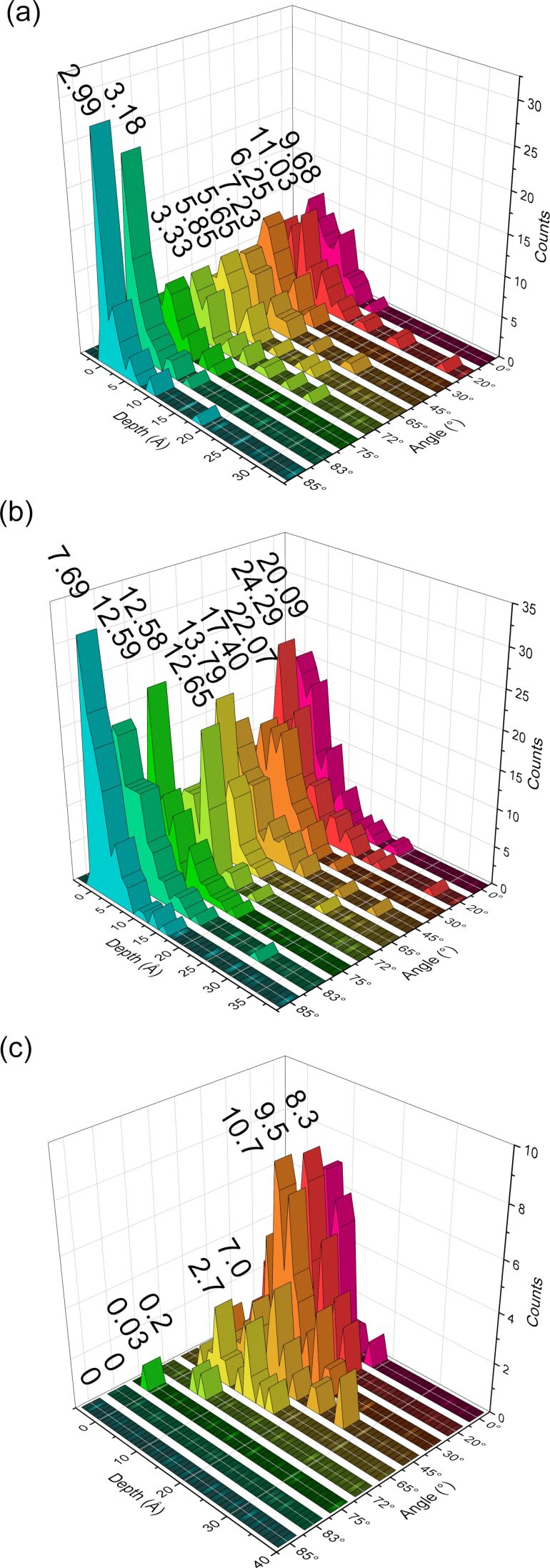
Distribution of implanted contaminants, (a) oxygen, (b) hydrogen and (c) argon, with respect to the selected angles. The values above the peaks represent the mean implantation depth. Different colours are used for different angles to increase the readability of the plots.

**Figure 14:**
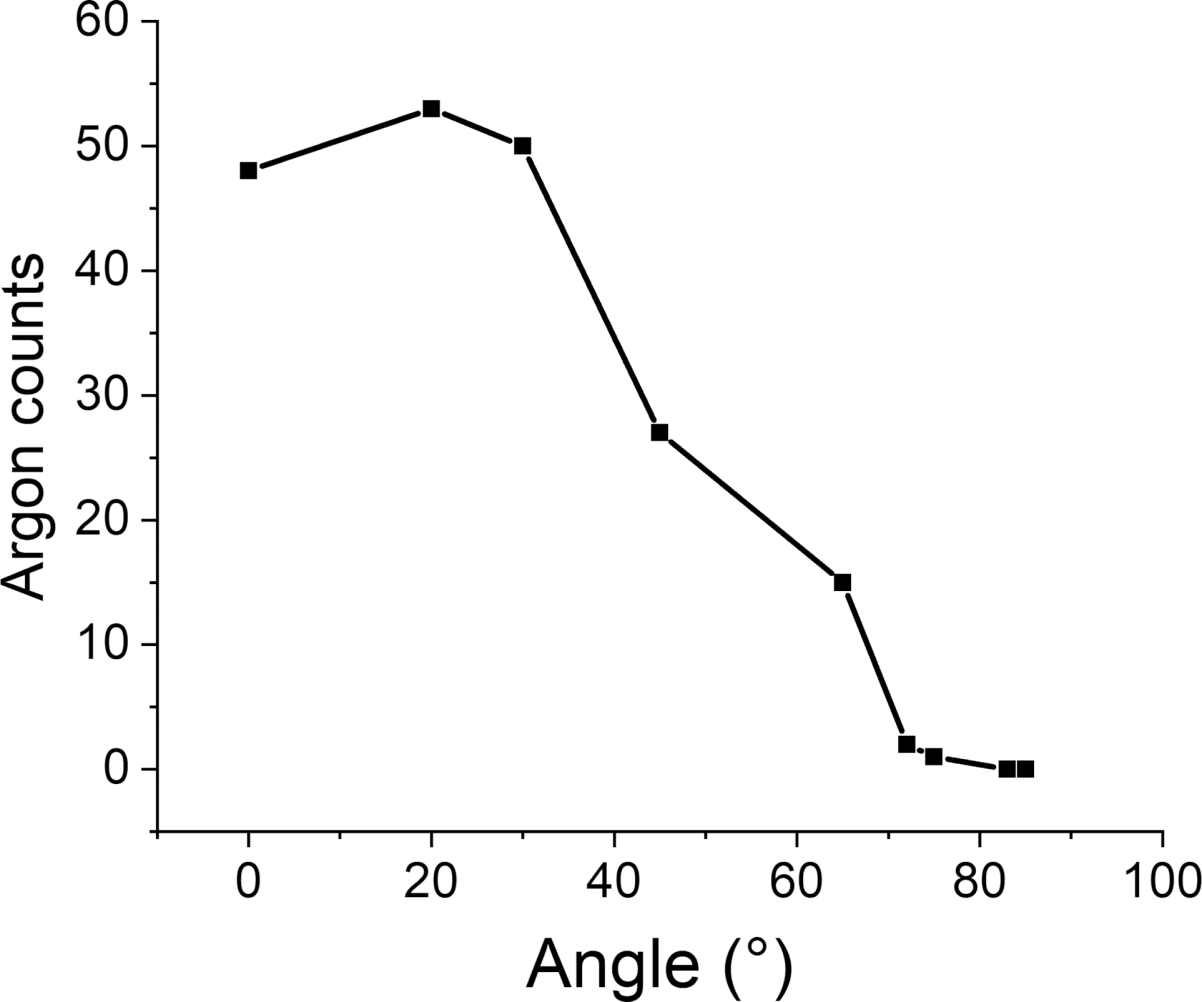
Number of argon atoms retained in the sample, regardless of the depth of implantation, with respect to the angle.

To quantify the impact of contaminants on the irradiation process, we looked at the distributions of implanted argon particles and compared them to the distribution of contaminants for each incidence angle. The result of these comparisons can be found in Figure 13. In Figure 14, the total number of retained argon atoms in the sample is plotted as a function of the angle. At normal incidence the Ar implantation depth is the highest and it decreases with the incidence angle. For angles above 80°, argon ions are not implanted at all into the sample; they are either backscattered or ejected from the sample during subsequent bombardments. Like the contaminant distribution, we can see some implantation depth deeper than the depth of the amorphous slab.

Furthermore, the mean implantation depth of hydrogen is twice as deep as that of oxygen. This behaviour can be explained by two factors: (i) Hydrogen atoms are involved twice as often as oxygen in displacements, due to the number of atoms of each species in the water molecule. This is true in the early stage of the sputtering process since the water molecules are intact and, therefore, the collision will either sputter or fragment the molecules. During latter events, hydrogen and oxygen might be pushed into the sample. At higher fluences, where most of the hydrogen atoms are dissociated from oxygen atoms, the probability of hydrogen being involved in a collision cascade is twice as high as that for oxygen, since the ratio between the two species stays approximately constant throughout the process. (ii) Since hydrogen is lighter than oxygen, the particle can easily channel through the silicon lattice and penetrate deeper into the sample. This phenomenon can happen if the hydrogen is impacted at the correct angle, either by initial collisions with argon ions or by recoil atoms during the collision cascade. This will allow it to travel in a straight path through the silicon lattice, reaching and causing damage in deeper regions.

Overall, the contaminant will be displaced into the amorphous region of the sample, causing this region to be thicker than that of the pristine sample. This effect can impact the preparation of samples such as ultrathin lamellae for TEM.

### Reaction products

**Figure 15:**
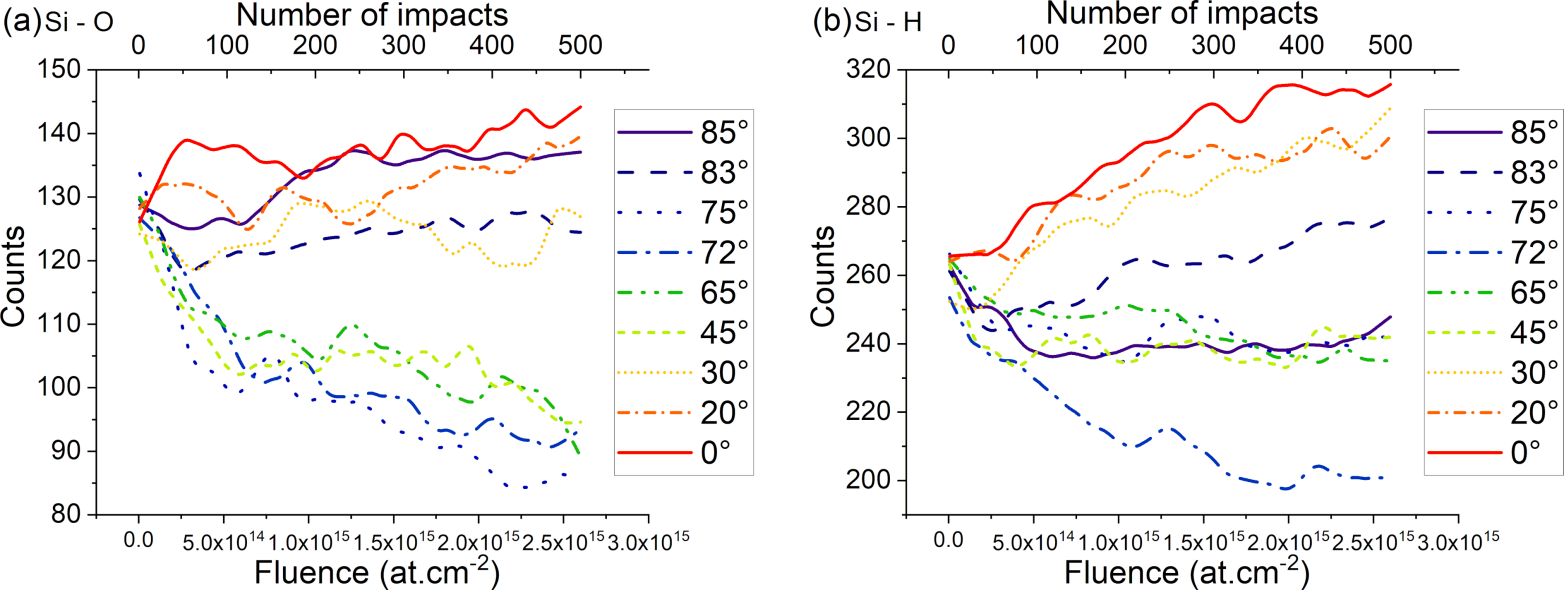
Evolution of the Si–O (a) and Si–H (b) products with respect to the fluence and the angle.

Water molecules are either fragmented or sputtered by ion bombardment so that oxygen and hydrogen atoms are implanted into the sample or sputtered. The implanted or adsorbed species and fragments will interact with the silicon atoms of the sample, forming reaction products, which contributes to the amorphization of the sample surface. These products can range from simple (e.g., Si–O or Si–H pairs) to complex (e.g., Si–H_3_–O products at higher incidence angles) products and are found near the surface region. Since the fragments are highly displaced in the near-surface, amorphous region and are continuously displaced by further argon impacts, the reaction products continuously change with fluence. In Figure 15, we showed the two most common products (i.e., Si–O and Si–H) and plotted the results with a sliding mean averaged over 50 points to get the trend and the evolution with respect to the incidence angle and the fluence. We observe a clear trend with a progressive reduction in the Si–O and Si–H counts for intermediate angles. Yet, at either low angles (0° – 30°) or high angle (>80°) we observe that the counts remain high and in the same order of magnitude. Hence, the implanted O and H atoms are either replaced or remain trapped in the amorphous layer. Most of the water molecules will be sputtered at higher incidence angles. The implanted species will tend to significantly move inside the amorphous region. This behaviour is due to either collisions displacing large amounts of atoms in the amorphized region, or to relaxation of previous displacement cascades. Due to the strong interaction between silicon and oxygen particles, Si–O products tend to be sputtered as a cluster or the oxygen migrates into the amorphous region.

The other relevant reaction products (e.g., Si–OH, Si–H_2_, and Si–O_2_) are displayed in Supporting Information File 1. In addition, a graph with the raw counts is displayed to give a better picture of the evolution of the reaction products.

### Sputtering yields and sputtered clusters

**Figure 16:**
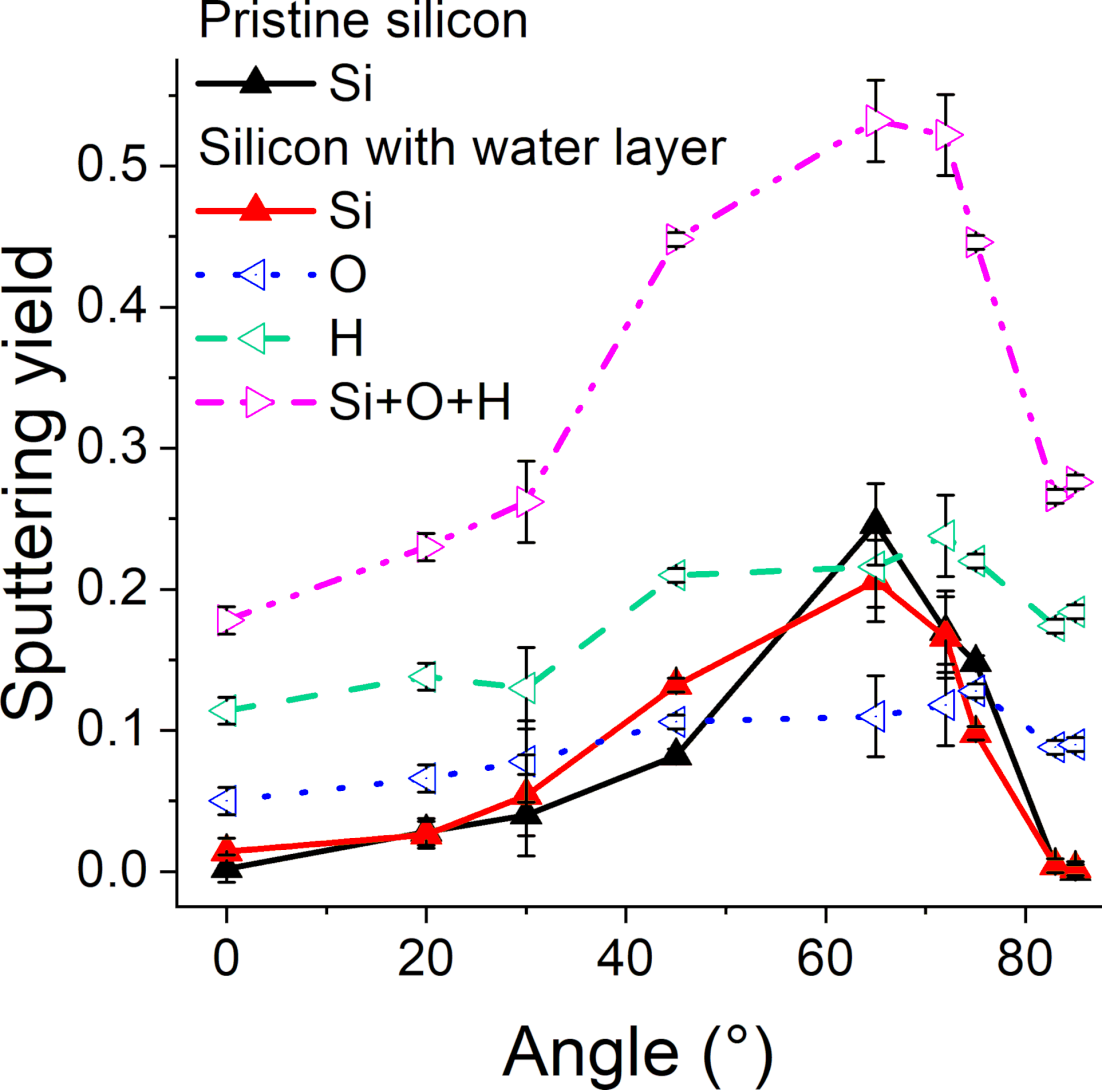
Sputtering yields of pristine and contaminated samples with respect to the incidence angle. For the sputtering yields of the contaminated sample, a distinction between partial Si, O, and H sputtering yields are shown in red, blue, and green, respectively, and the total sputtering yield is shown in magenta.

Sputtering yields are important for experiments as they define how quickly a sample is eroded. Here, we compare the sputtering yields of pristine and contaminated samples averaged over 500 impacts (Figure 16). No significant difference can be observed between silicon sputtering yields coming from pristine and contaminated samples. As expected, both sputtering yields increase with an increasing incidence angle to reach a maximum of approx. 65°. For partial sputtering yields of hydrogen and oxygen, the curves are flatter and reach their maximum values for incidence angles of approx. 70° (i.e., for higher grazing incidence angles than those of silicon). The total sputtering yield of the contaminated sample (i.e., the sum of silicon, oxygen, and hydrogen partial sputtering yields) is much higher than that of the pristine sample, showing that water molecules are quite easily sputtered.

**Figure 17:**
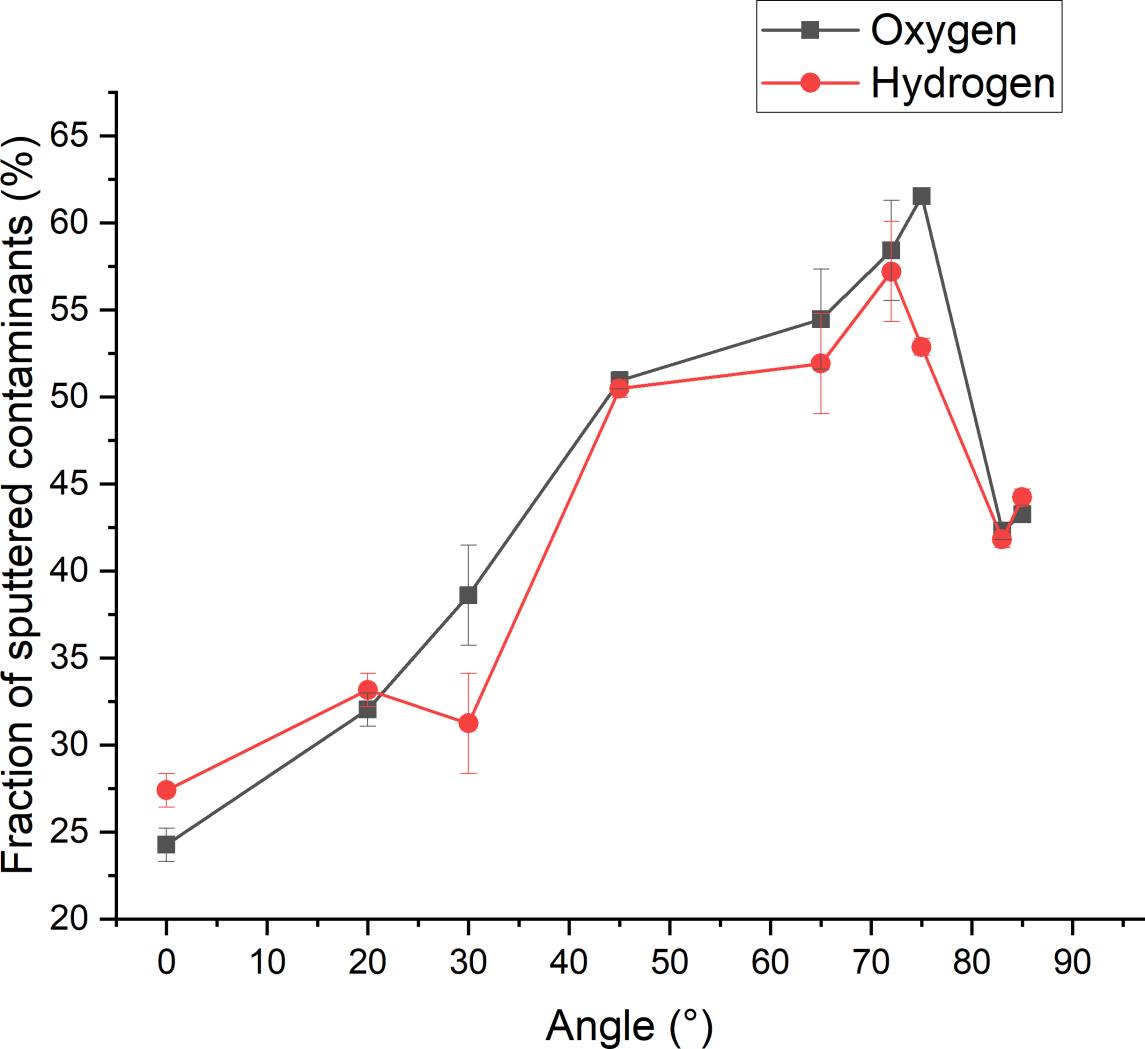
Fraction of the contaminants sputtered after 500 impacts with respect to the angle. The values are represented as the percentage of total particles removed by counting the contaminant particles in the initial sample and deducting the counts in the final steps for each angle.

A second parameter of interest related to sputtering is the sputtered fraction of hydrogen and oxygen atoms (Figure 17). At normal incidence, approx. 25% of the hydrogen and oxygen atoms are sputtered, meaning that by far the largest fraction is implanted and only a few atoms stay on the sample surface. This can be explained by the fact that the velocity vector of argon ions is oriented straight into the sample, and this orientation is maintained for the energy transfer to the target atoms. Interestingly, the partial sputtering yields of hydrogen and oxygen atoms are the highest at incidence angles between 70 and 80° (i.e., above angles where the silicon sputtering yields are the highest), leading to the sputtering of approx. 75% of the adsorbed molecules. At these angles, most of the argon ions are backscattered. However, the energy transfer is still high enough to fragment the water molecules and induce the sputtering of hydrogen and oxygen atoms. Hence, if the implantation of surface contaminations is to be minimized in experiments, the incidence angle should be chosen in this range.

In the current simulations, the water layer was added before the argon irradiation was started. When renewing the water layer during the irradiation process, as it happens during experiments, the partial sputtering yield of silicon might be lowered by the presence of the surface contamination, at least if the contamination level is above a critical threshold.

In order to study in our simulations the evolution of a given sample under continuous irradiation, including the effect of water contamination, we kept the energy at 100 eV and argon ions were bombarding the sample one after the other, in a dynamic sputtering mode. This differs from the literature where monatomic and cluster bombardment at high energies can be found (e.g., [66–68]). These studies also use single ion bombardment and no dynamic sputtering. Yet, despite these differences, we observed a similar range of damage and amorphization as in [68], which in our study was increased by the presence of water molecules. Furthermore, the predicted sputtering yields in [69] for energies between 200 and 500 eV at normal incidences using an angular distribution of +/−5° are bigger than those in our observations, which at normal incidence give a very low sputtering yield. Energies of approx. 100 eV are closer to the sputtering threshold, and the incidence angle plays a critical role as our data, at slightly off-normal conditions, matches their predicted yields.

**Figure 18:**
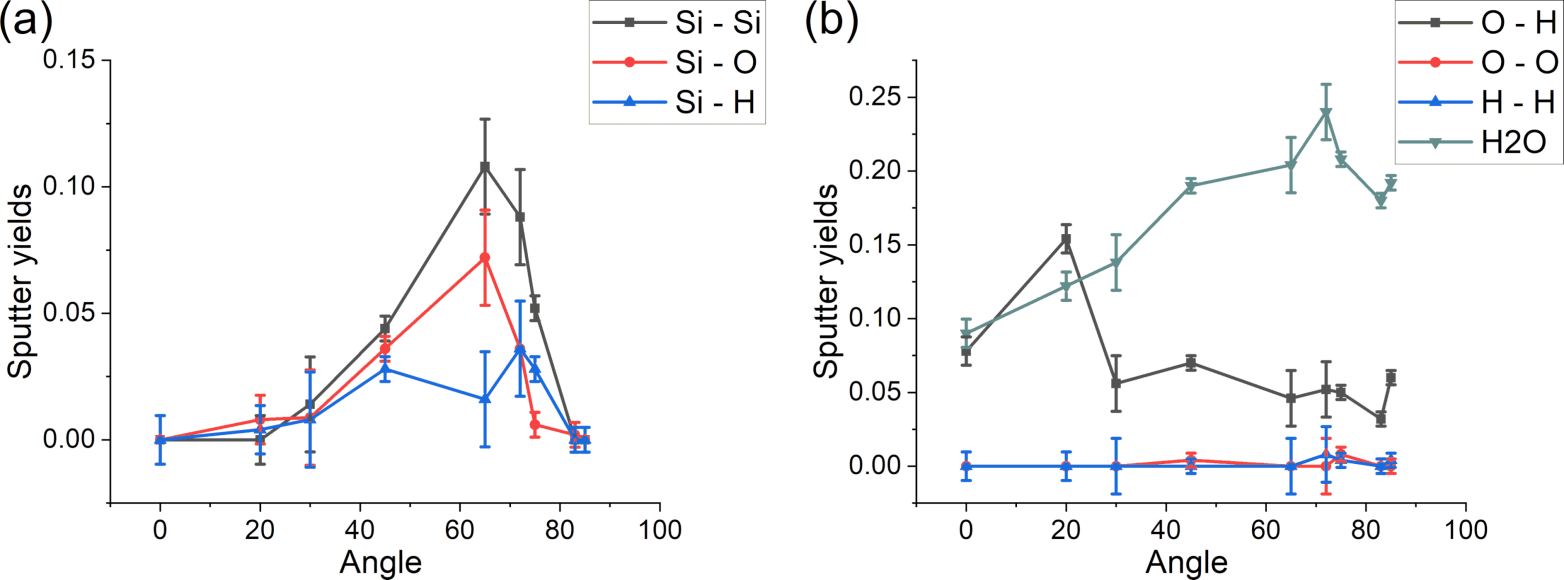
Sputtering yields for (a) silicon-related and (b) oxygen-related clusters.

A third parameter of interest is the distribution of the sputtered clusters. Overall, several cases are observed: (i) an atom is sputtered alone, (ii) several atoms are sputtered during the same collision cascade independently of one another, and (iii) a cluster of atoms is sputtered. In Figure 18 the evolution of the sputtered clusters is plotted with respect to the incidence angle. The highest sputtering yield is observed for intact water molecules, usually occurring in the first simulations when the water layer is relatively undamaged. At higher incidence angles, the amount of water molecules which is sputtered intact is the highest: since the argon ion is at grazing incidence, the energy transfer is efficient for molecular lift-off and far less for bond breaking, which predominantly happens towards normal incidence. This explains also the increased amount of sputtered O–H clusters at smaller angles where the argon ions more aggressively damage the water layer, increasing the probability of water fragmentation. For silicon, the most common sputtered cluster is Si_2_. Its sputtering yield follows the same trend of Si. The second most common fragment for silicon is SiO, which is especially stable due to the nature and the strength of the Si–O bonds. Finally, there are low counts for Si–H and more complex clusters. They represent an almost negligible fraction of the total counts of the sputtered particles.

**Figure 19:**
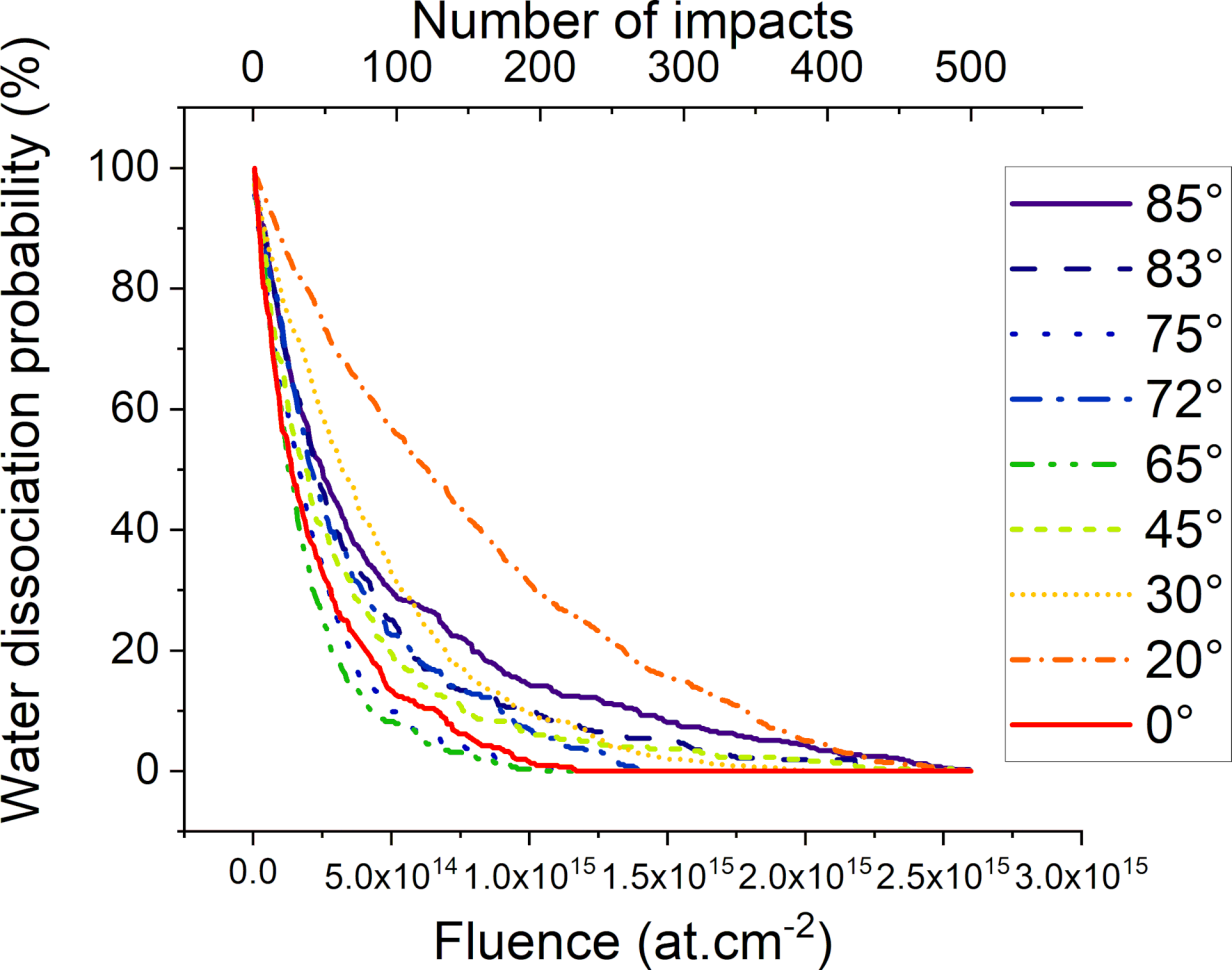
Graph showing the probability to dissociate at least one water cluster per impact, with respect to angle and fluence. In some cases, one collision could dissociate several clusters of water.

For most simulations there is no intact water molecule remaining at the end of the sputtering events. This is shown in Figure 19 which describes the number of intact clusters with respect to fluence or to the number of impacts, respectively. From this observation we can deduce that most damages occur during the first irradiation simulations, either by sputtering or fragmentation. Once most of the water molecules have been fragmented, the atomic mixing is facilitated by the presence of fragments in the amorphous region. Furthermore, at higher incidence angles we observe an increased rate of water dissociation.

## Conclusion

In this work, we analysed the influence of the incidence angle on 100 eV sputtering processes. We used MD simulations for irradiation with argon, with fluences up to 2.6 × 10^15^ ions/cm^2^ and ions of silicon samples with a clean surface and covered with a monolayer of water molecules. Contaminants adsorbed on the sample surface amplify the damage done by the argon ions. A methodology based on the modification of bond lengths has been developed to characterize the irradiation-induced damage. Three regions have been identified in the sample: (i) a totally amorphous layer closer to the surface, (ii) a transitive, partially amorphized layer presenting defects, and (iii) the crystalline region, which remains unaffected underneath by ion irradiation. Closer to grazing incidence (e.g., above 75°), argon implantation becomes sparse; however, fragmentation and sputtering of water molecules reach maximum values. Hence, sputtering yields and molecular fragmentation show that specific angles can be selected to reduce the mixing of contaminants into the sample in ultralow energy sputtering. Compared to the pristine sample, the mixing of hydrogen and oxygen atoms into the sample increases the overall degree of amorphization. Furthermore, the water layer on the silicon surface was prepared before ion irradiation and not renewed during the milling process. However, in experiments, contaminations are continuously adsorbed on the sample surface, leading to a renewal of the contamination layer. Furthermore, at higher impact energy the implantation depth of the ions is increased, leading most likely to a different amorphization process. Both aspects will be explored in future studies.

## Supporting Information

File 1 Additional figures and tables with DFT data fitting plots, g(r) per slab and grouping details, reaction products, and ReaxFF potential.
