# Supplementary material for: Influence of water contamination on the sputtering of silicon with low-energy argon ions investigated by molecular dynamics simulations
Source: Beilstein J Nanotechnol. 2022 Sep 21;13:986–1003. doi: 10.3762/bjnano.13.86 (PMC9520830; doi:10.3762/bjnano.13.86)
Supplement: File 1 — Additional figures and tables with DFT data fitting plots, g(r) per slab and grouping details, reaction products, and ReaxFF potential. [file Beilstein_J_Nanotechnol-13-986-s001.pdf]

BEILSTEIN JOURNAL OF NANOTECHNOLOGY

Supporting Information

for

Influence of water contamination on the sputtering of silicon with low-energy argon ions investigated by molecular dynamics simulations

Grégoire R. N. Defoort-Levkov, Alan Bahm and Patrick Philipp

Beilstein J. Nanotechnol. **2022,** *13,* 986–1003. doi:10.3762/bjnano.13.86

Additional figures and tables with DFT data fitting plots, g(r) per slab and grouping details, reaction products, and ReaxFF potential

License and Terms: This is a supporting information file under the terms of the Creative Commons Attribution License (https://creativecommons.org/licenses/by/4.0). Please note that the reuse, redistribution and reproduction in particular requires that the author(s) and source are credited and that individual graphics may be subject to special legal provisions.
The license is subject to the *Beilstein Journal of Nanotechnology* terms and conditions: (https://www.beilstein-journals.org/bjnano/terms)

Index

DFT data fitting plots

Figure S1. Plot of Ar–Si interactions with fitting parameters D_0, α, and r_0 used in the LAMMPS script to output the potential. The full potential is displayed.

Figure S2. Plot of Ar–Si displaying the expected upper energy of the incoming argon ions (100 eV).

Figure S3. Plot of Ar–Si displaying a focused part, emphasising the potential well describing the interatomic forces (switch from repulsive to attractive part of the potential).

Figure S4. Plot of Ar–Si displaying an extremely focused part, further increasing the observation of the potential well and the minor discrepancies between the fit and the potential from DFT.

Figure S5. Plot of Ar–O interactions with fitting parameters D_0, α, and r_0 used in the LAMMPS script to output the potential. The full potential is displayed.

Figure S6. Plot of Ar–O displaying the expected upper energy of the incoming argon ions (100 eV).

Figure S7. Plot of Ar–O displaying a focused part, emphasising the potential well describing the interatomic forces (switch from repulsive to attractive part of the potential).

Figure S8. Plot of Ar–H interactions with fitting parameters D_0, α, and r_0 used in the LAMMPS script to output the potential. The full potential is displayed.

Figure S9. Plot of Ar–H displaying the expected upper energy of the incoming argon ions (100 eV).

Figure S10. Plot of Ar–H displaying a focused part, emphasising the potential well describing the interatomic forces (switch from repulsive to attractive part of the potential).

Figure S11. Plot of Ar–Ar interactions with fitting parameters D_0, α, and r_0 used in the LAMMPS script to output the potential. The full potential is displayed.

Figure S12. Plot of Ar–Ar displaying the expected upper energy of the incoming argon ions (100 eV).

Figure S13. Plot of Ar–Ar displaying a focused part, emphasising the potential well describing the interatomic forces (switch from repulsive to attractive part of the potential).

G(r) per slab and grouping details

Figure S14. Plot of the detailed radial distribution functions for each slab, with a colour grading depending on the region for 100 eV, 0° bombardments at normal incidence, after 500 simulations.

Figure S15. Plot of the detailed radial distribution functions for each slab, with a colour grading depending on the region for 100 eV, 45° bombardments at a 45° incidence, after 500 simulations.

Figure S16. Plot of the detailed radial distribution functions for each slab, with a colour grading depending on the region for 100 eV, 75° bombardments at normal incidence, after 500 simulations.

Reaction products

Figure S17. Plot showing the raw counts for all the Si–H and Si–O products in the sample, with respect to incidence angle and fluence, with sliding mean curves averaged over 50 points to more clearly observe the trend.

Figure S18. Plot of the evolution of the Si–OH products in the sample with respect to angle and fluence. The Si–OH products remain marginal in counts in comparison to the Si–H and Si–O, and their trends mostly are statistical fluctuations. The data uses the 50 points sliding mean approach.

Figure S19. Plot of the evolution of the Si–O_2 products in the sample with respect to angle and fluence. The Si–OH products remain marginal in counts in comparison to Si–H and Si–O, and their trends mostly are statistical fluctuations. The data uses the 50 points sliding mean approach.

Figure S20. Plot of the evolution of the Si–H_2 products in the sample with respect to angle and fluence. The Si–OH products remain marginal in counts in comparison to Si–H and Si–O, and their trends mostly are statistical fluctuations. The data uses the 50 points sliding mean approach.

ReaxFF Potential

Table S1. Force field parameters, from "Oxidation of Silicon Carbide by O2 and H2O: A ReaxFF Reactive Molecular Dynamics Study, Part I" by David A. Newsome. et. al. - *J. Phys. Chem. C* 2012, 116, 30, 16111–16121.

DFT data fitting plots

Figure S1. Ar–Si potential, full potential.

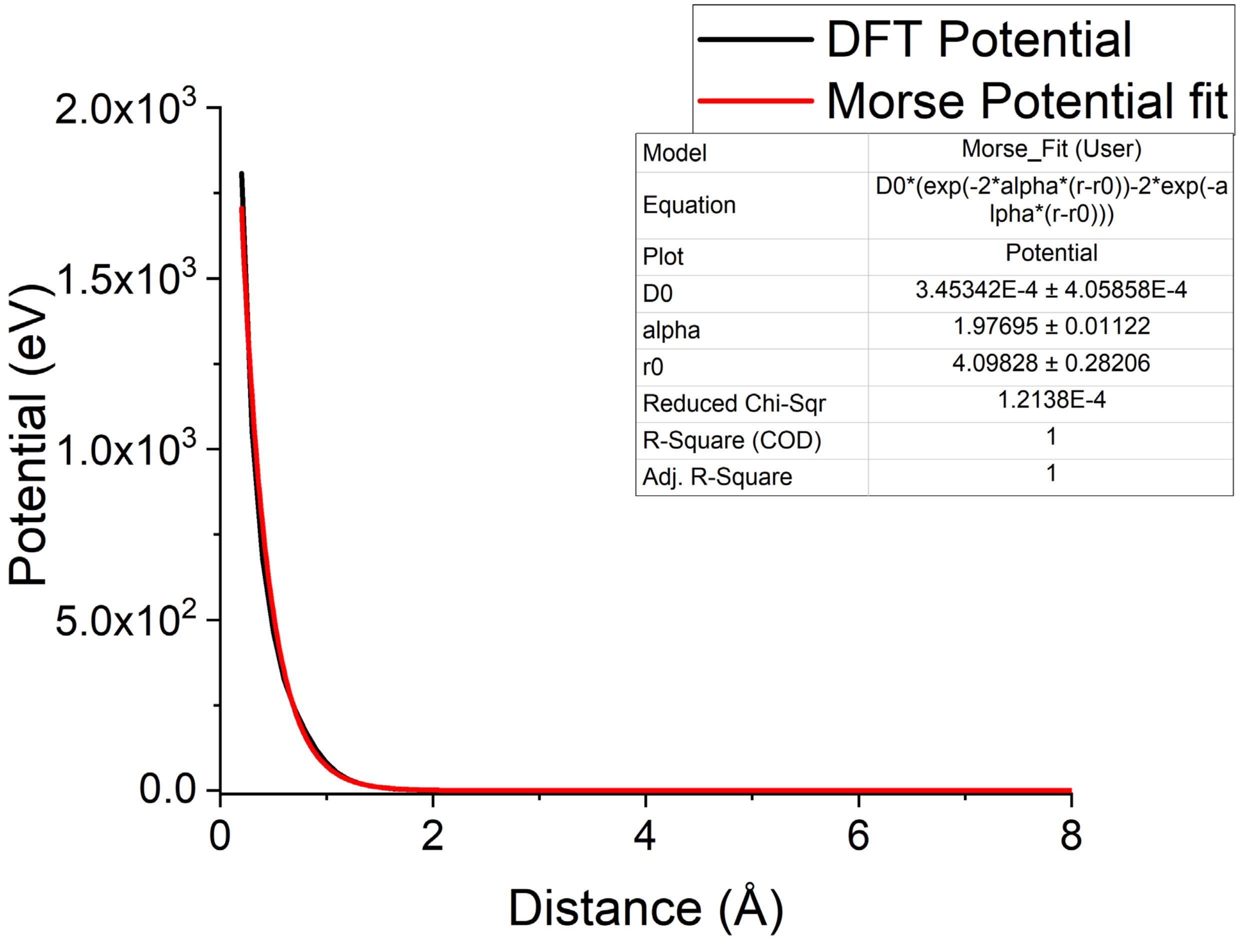

Figure S2. Ar–Si potential, 100 eV limit.

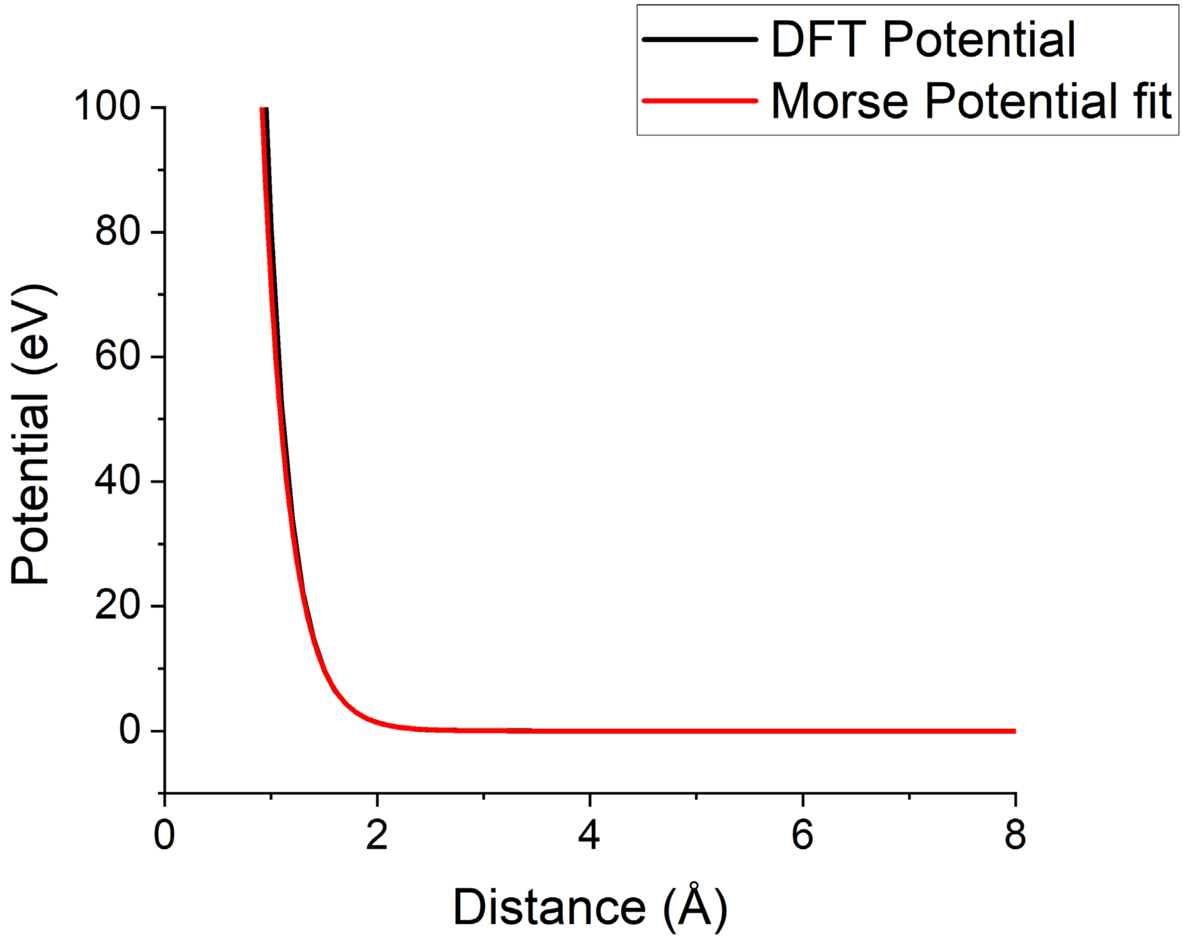

Figure S3. Ar–Si potential, focused part of the potential.

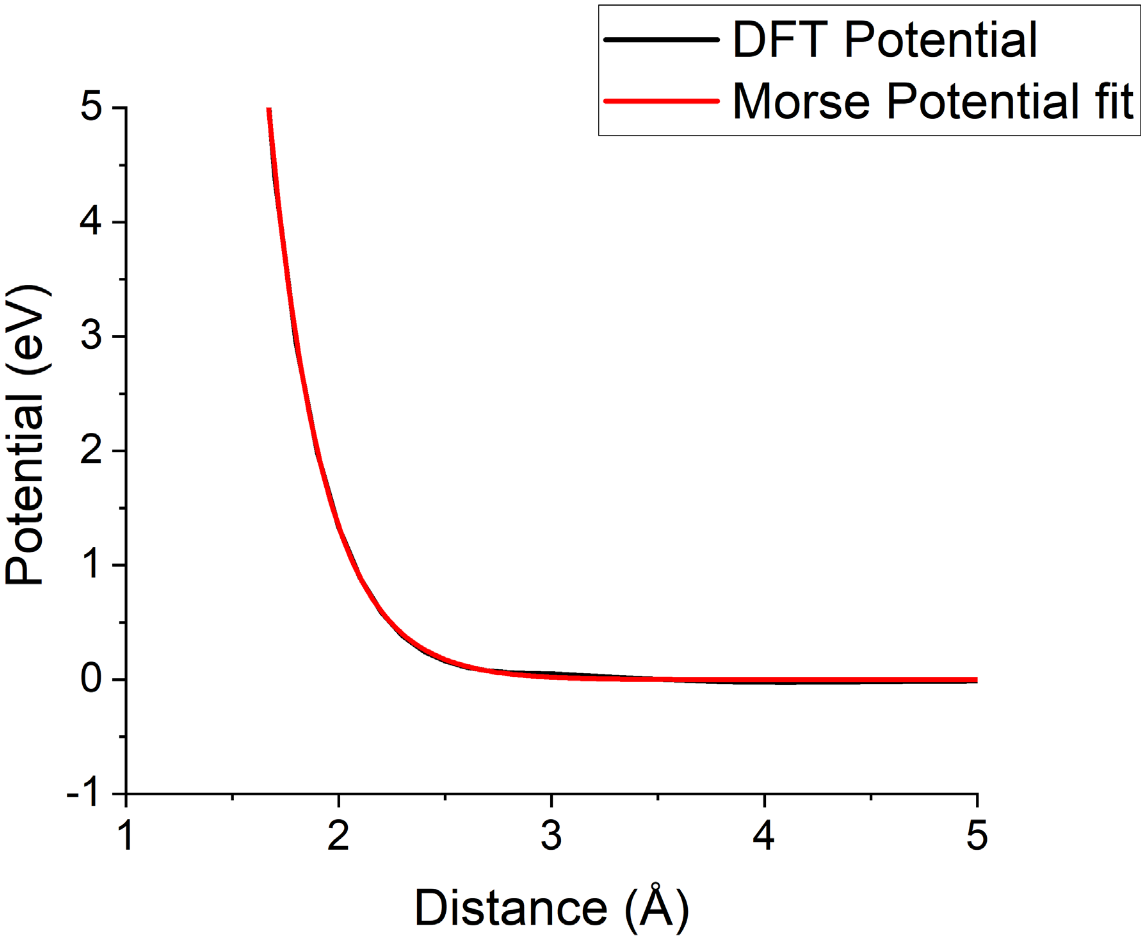

Figure S4. Ar–Si potential, extreme zoom.

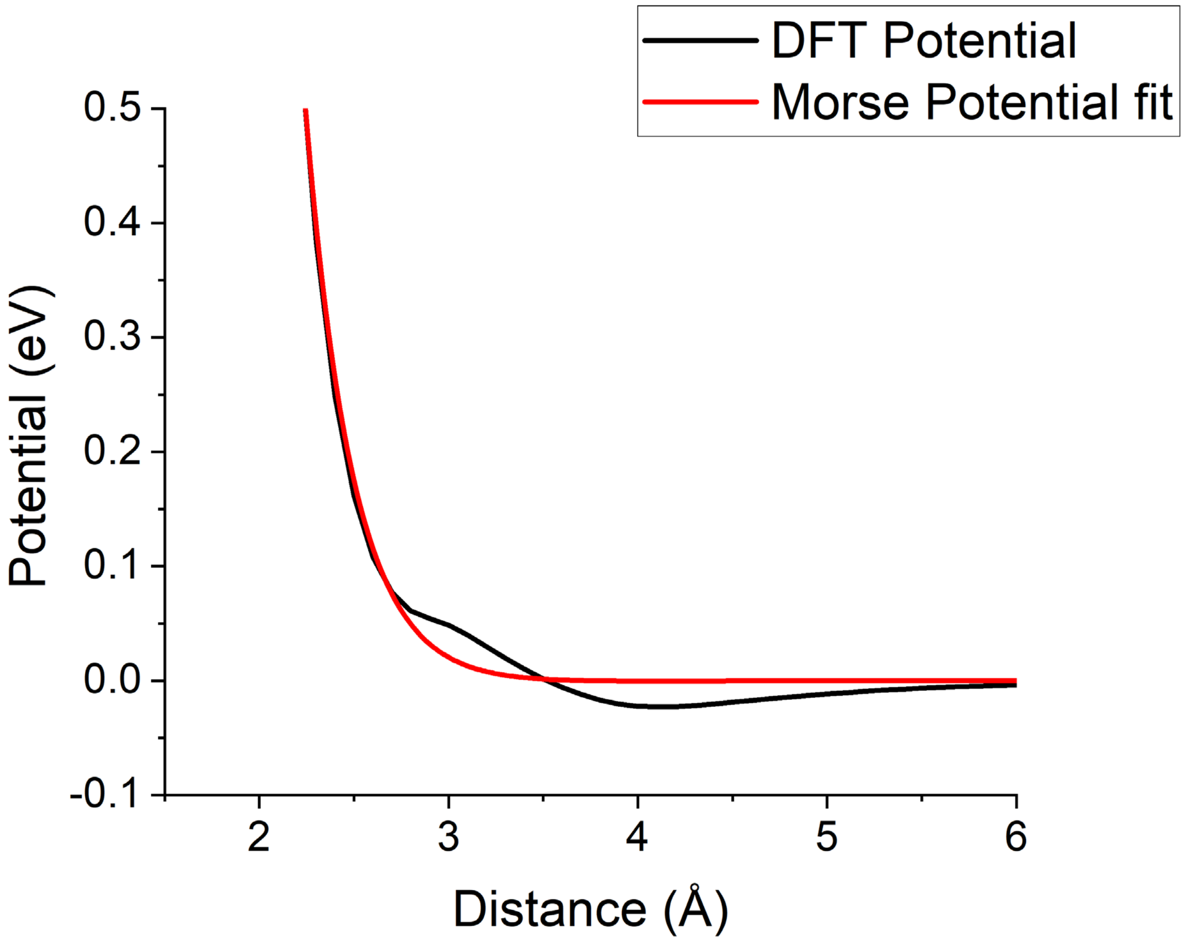

Figure S5. Ar–O potential, full potential.

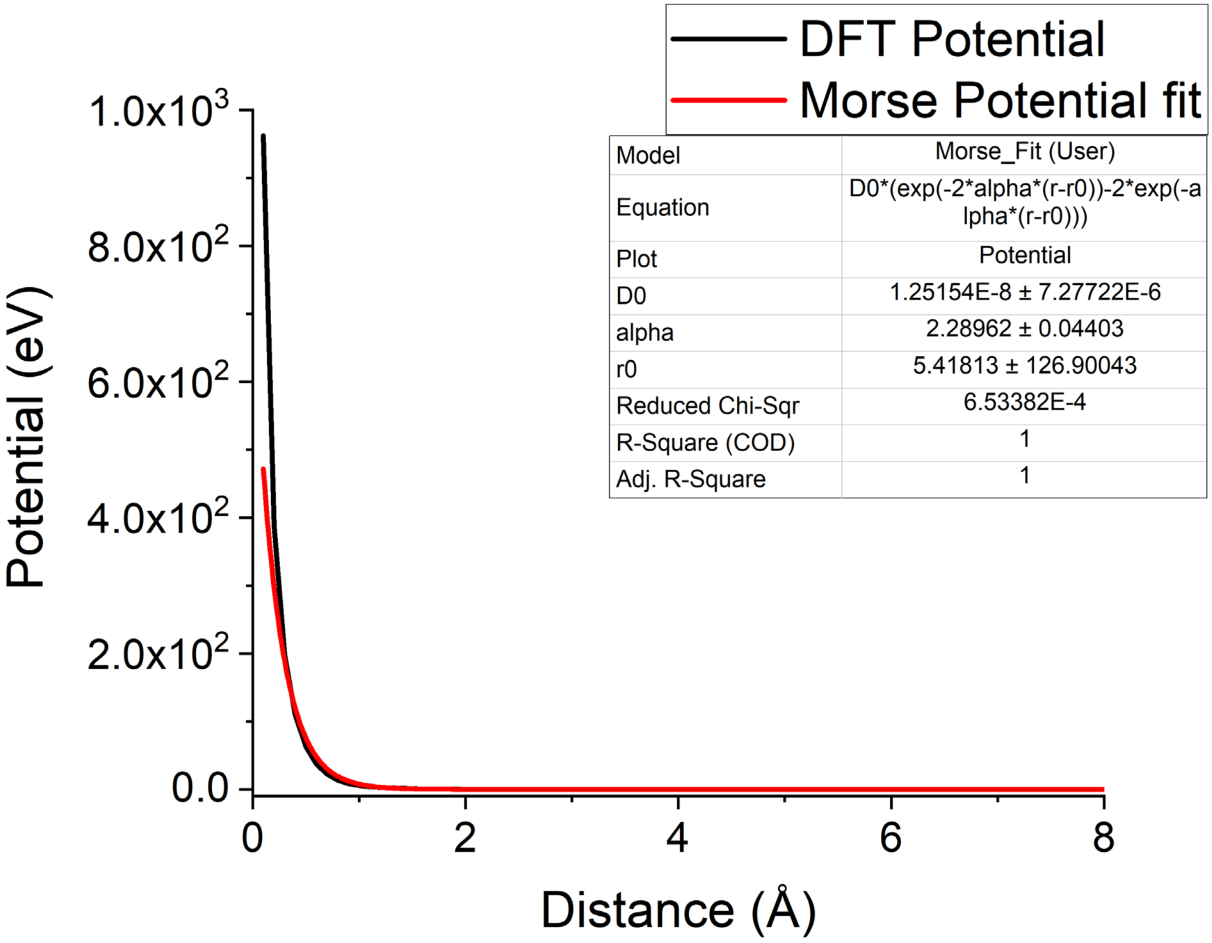

Figure S6. Ar–O potential, 100 eV limit.

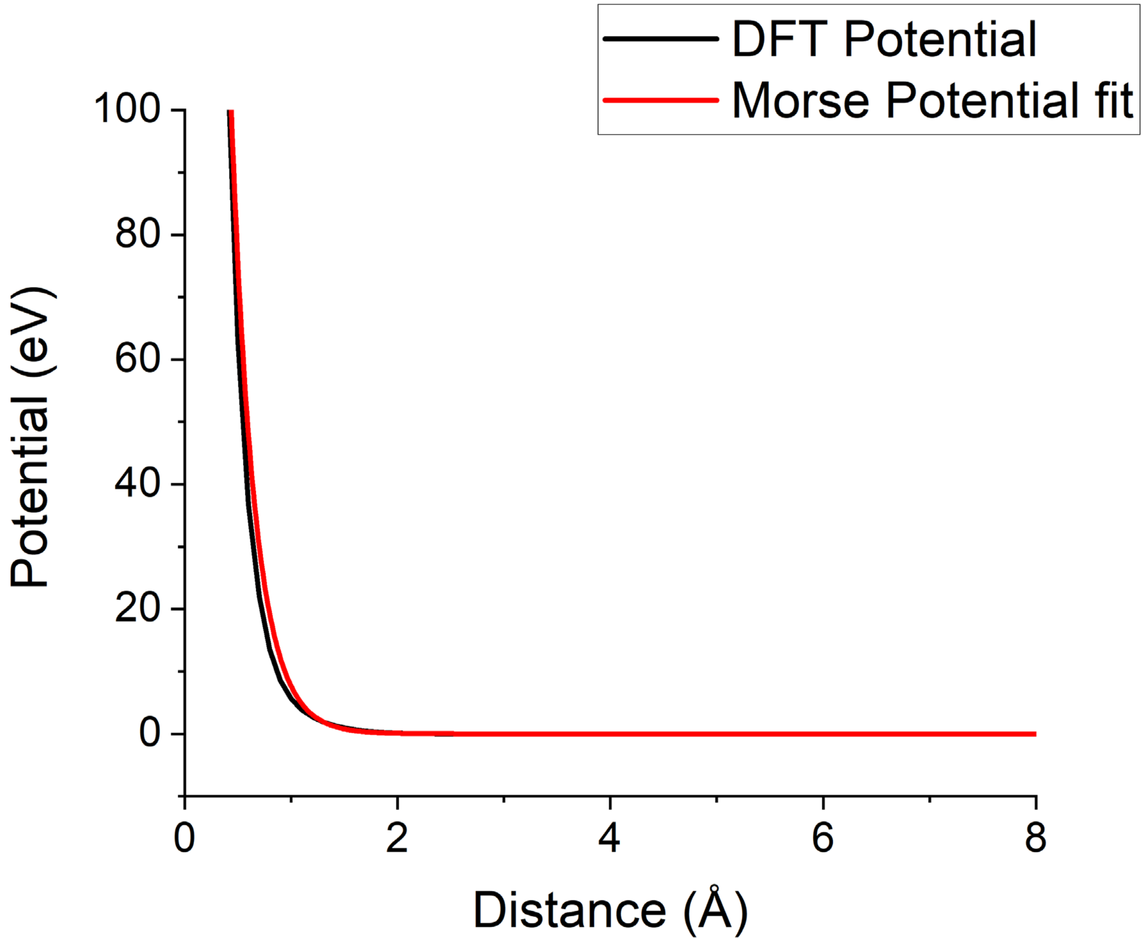

Figure S7. Ar–O potential, focused part of the potential.

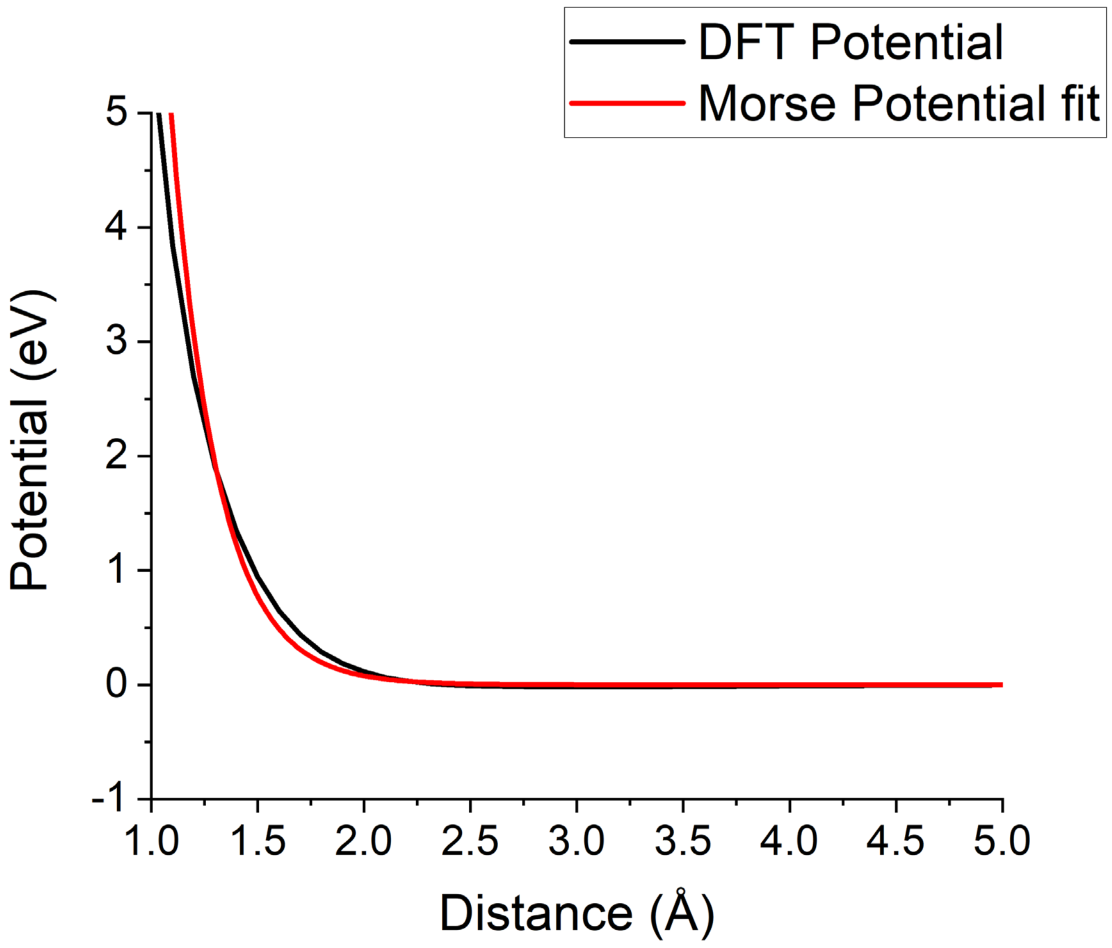

Figure S8. Ar–H potential, full potential.

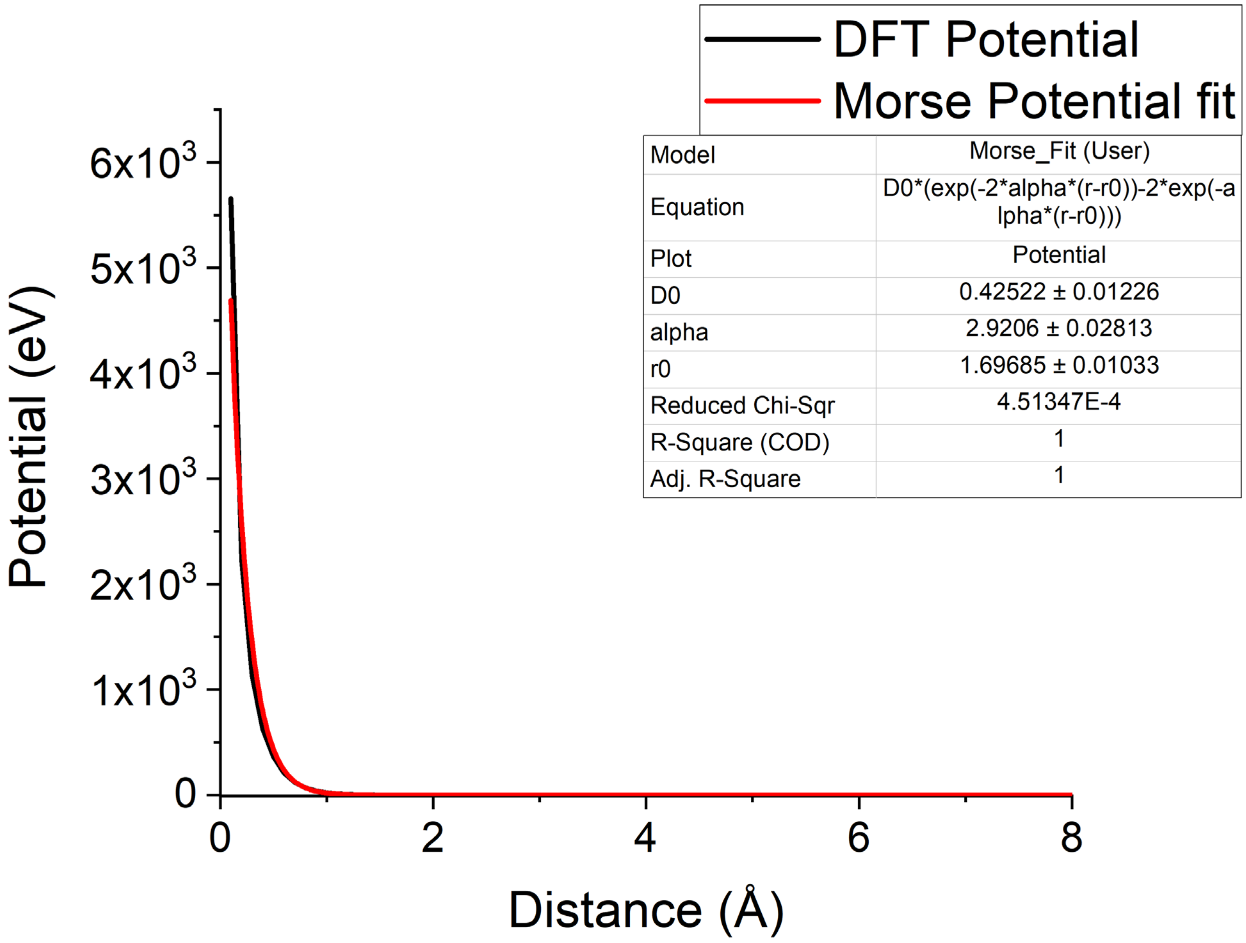

Figure S9. Ar–H potential, 100 eV limit.

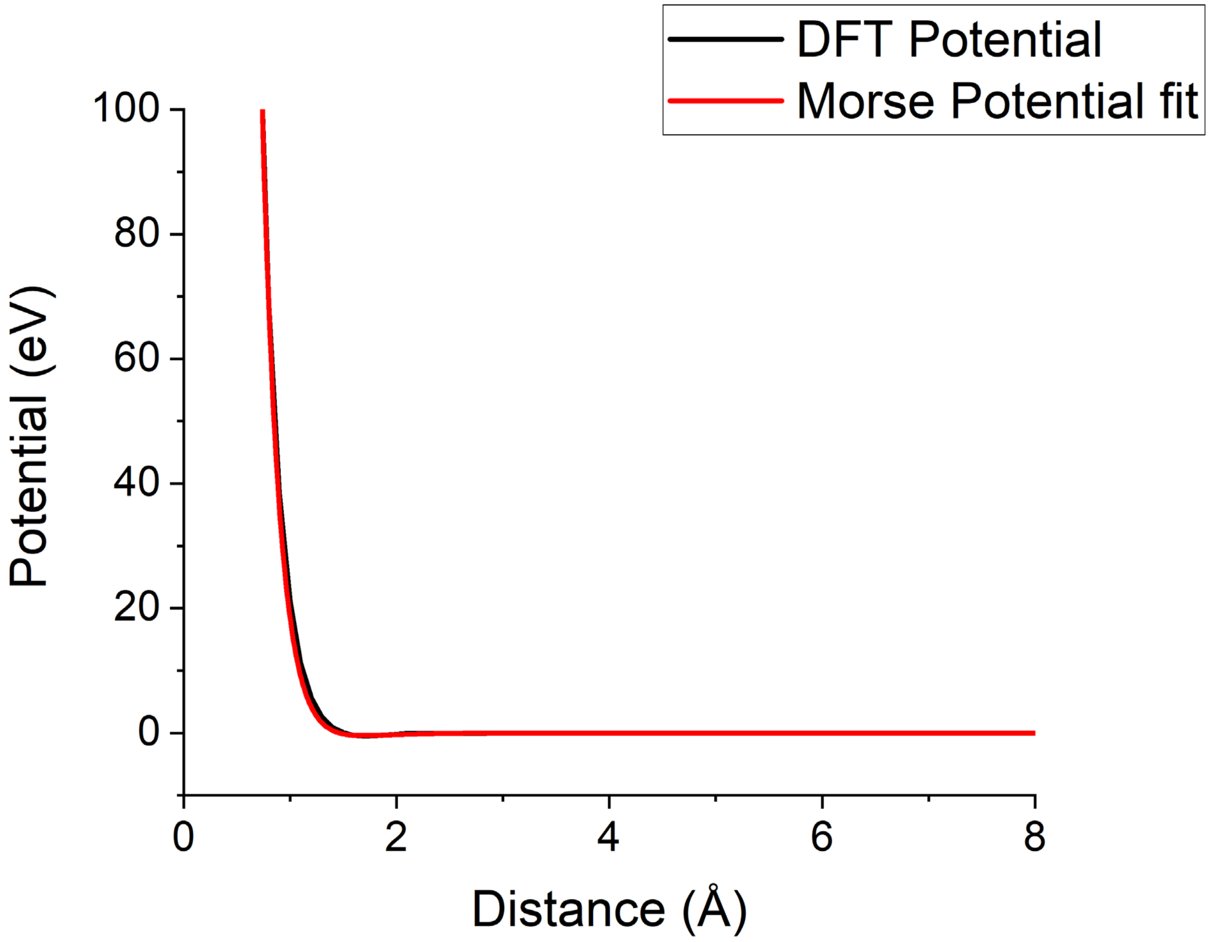

Figure S10. Ar–H potential, focused part of the potential.

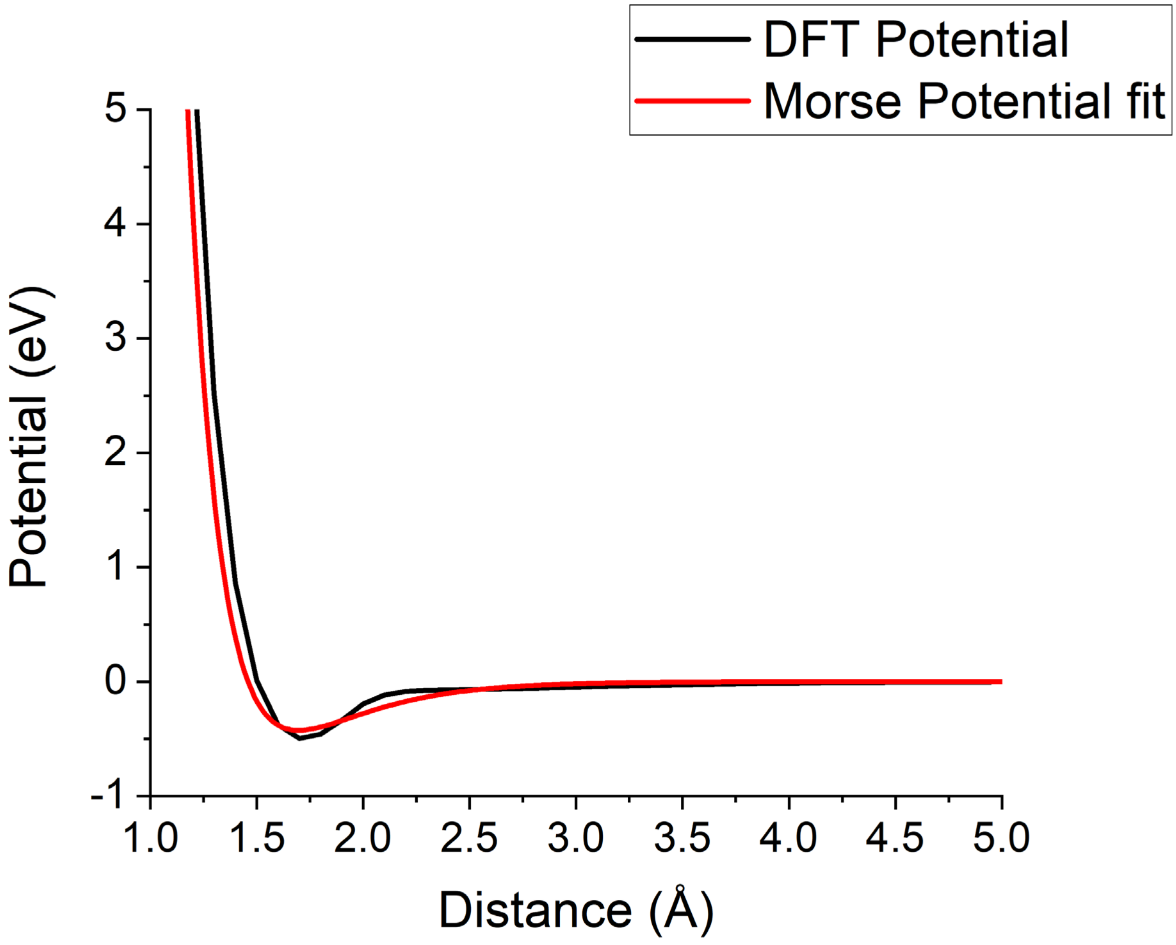

Figure S11. Ar–Ar potential, full potential.

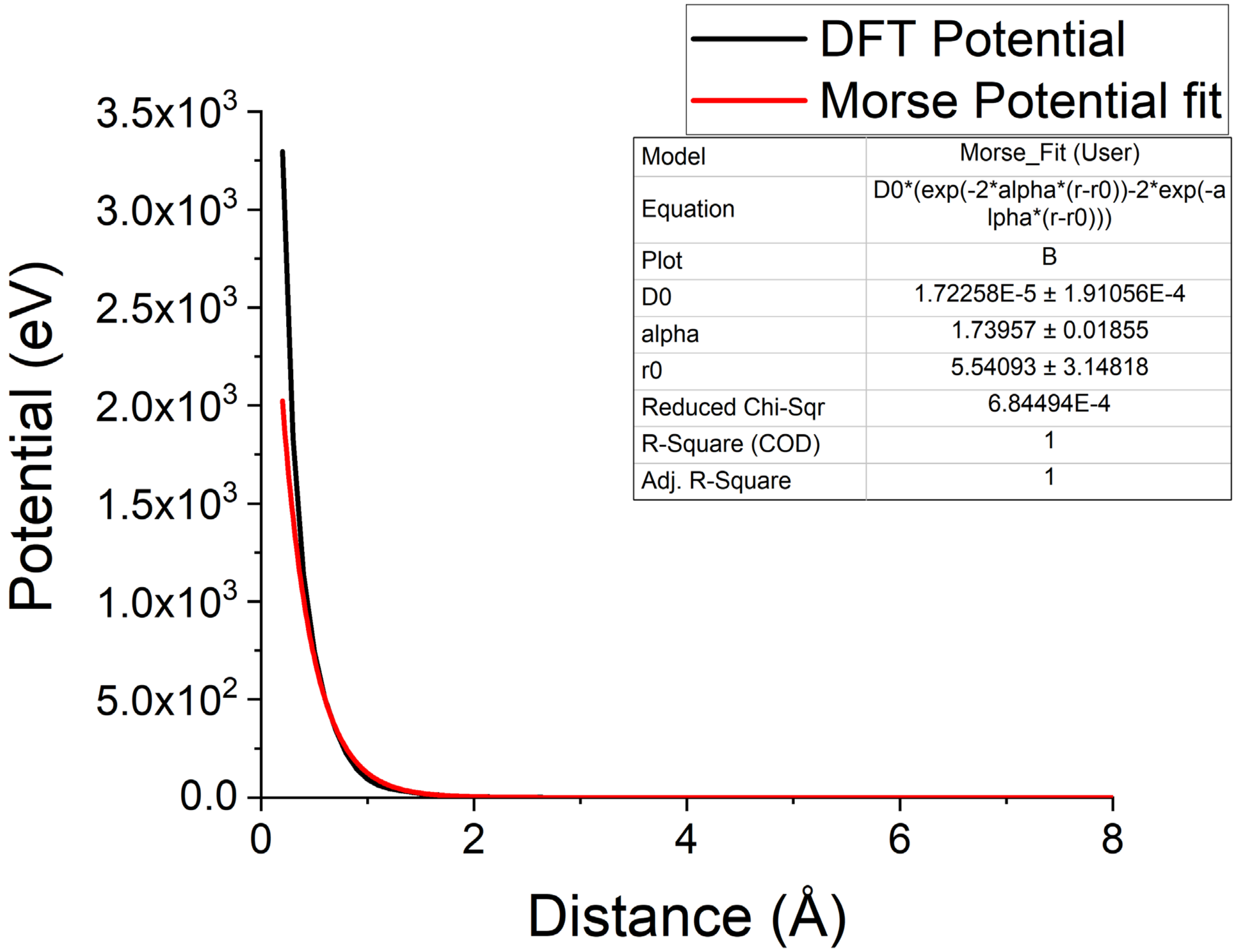

Figure S12. Ar–Ar potential, 100eV limit.

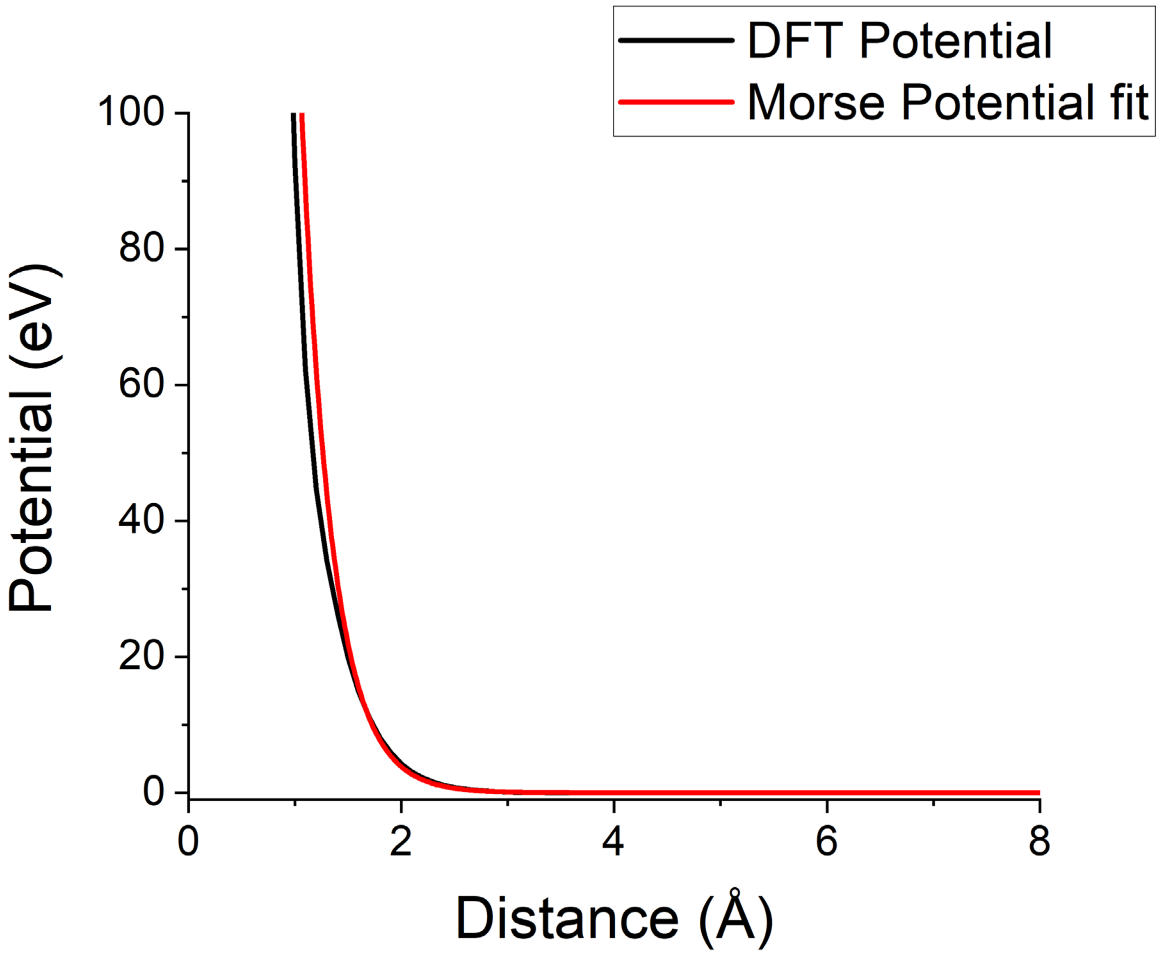

Figure S13. Ar–Ar potential, focused part of the potential.

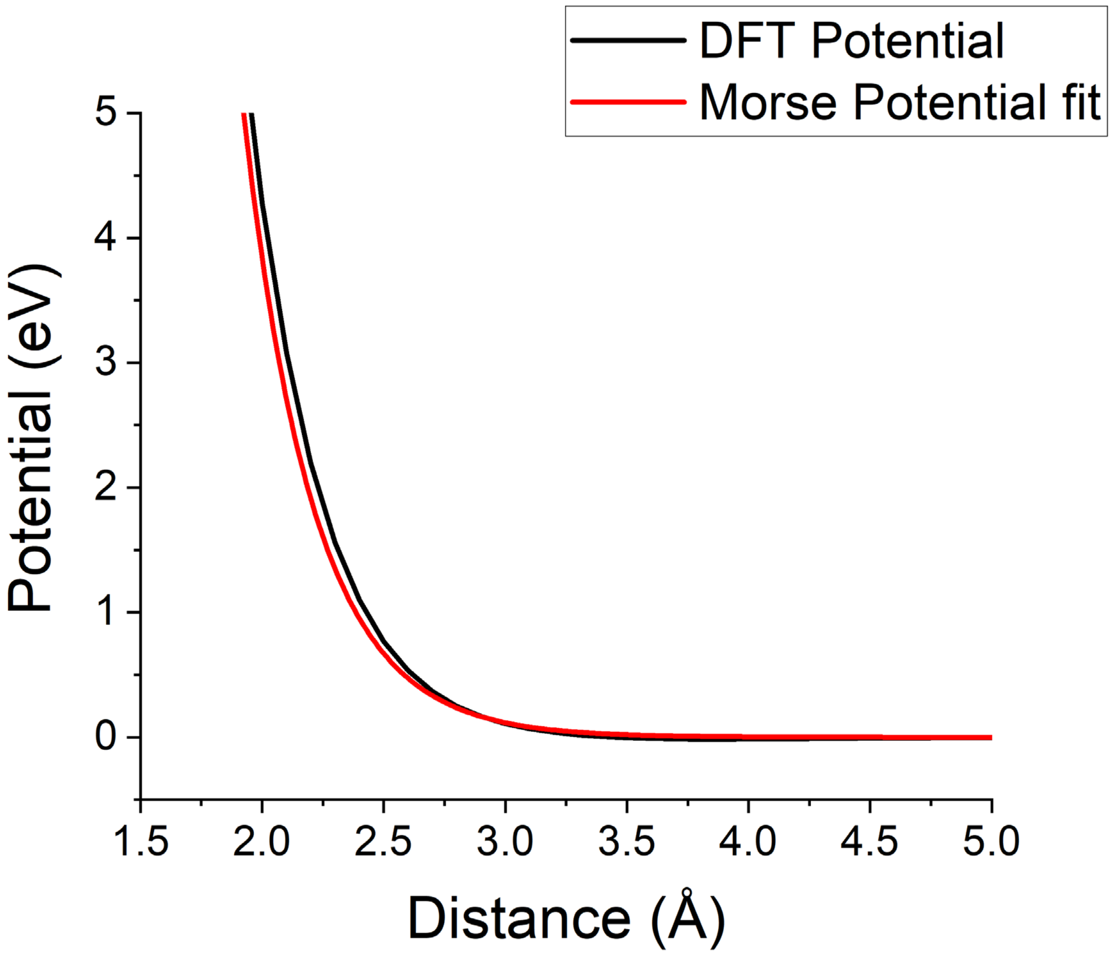

G(r) per slab and grouping details

Figure S14. Detailed g(r) for each slab for 100 eV, 0°, and 500 bombardments.

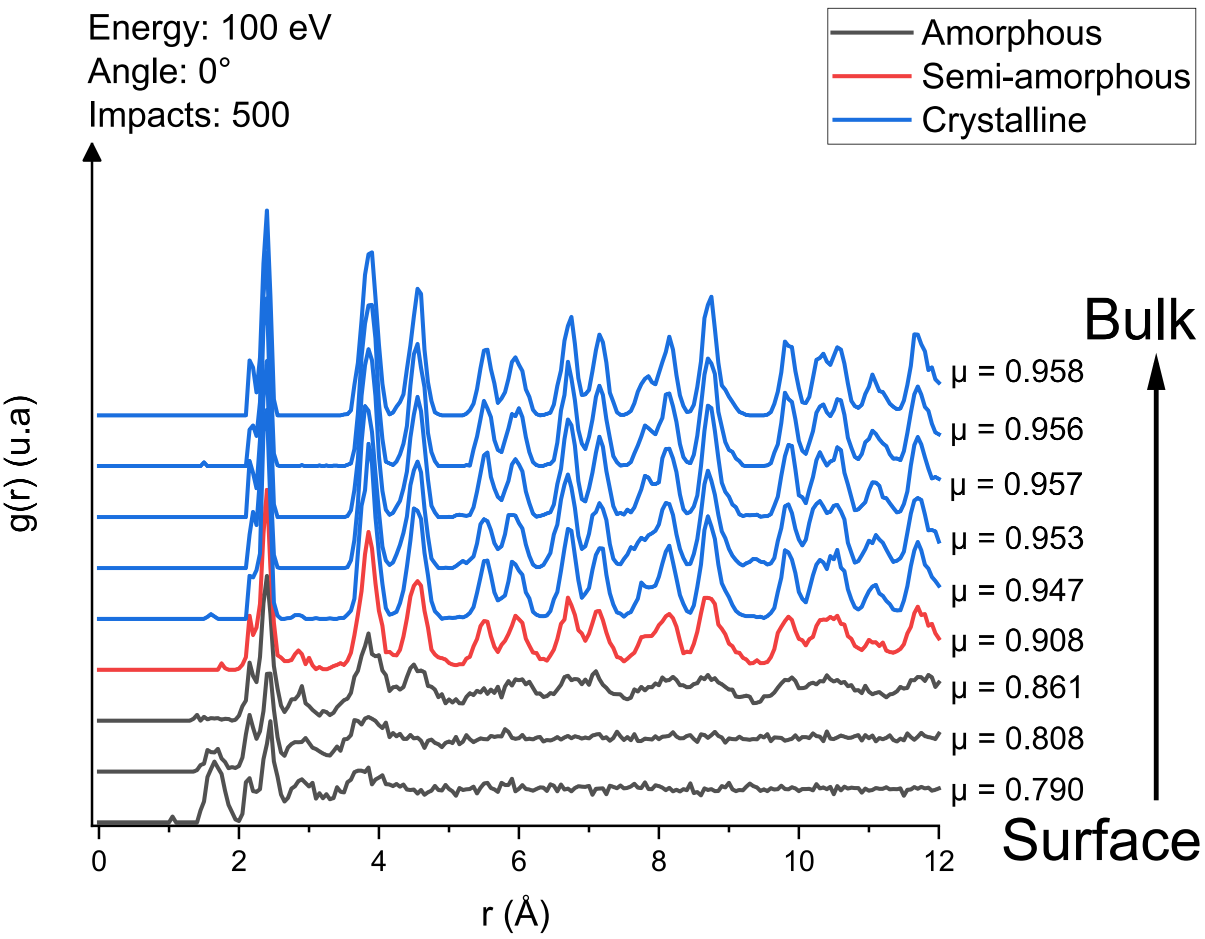

Figure S15. Detailed g(r) for each slab for 100 eV, 45°, and 500 bombardments.

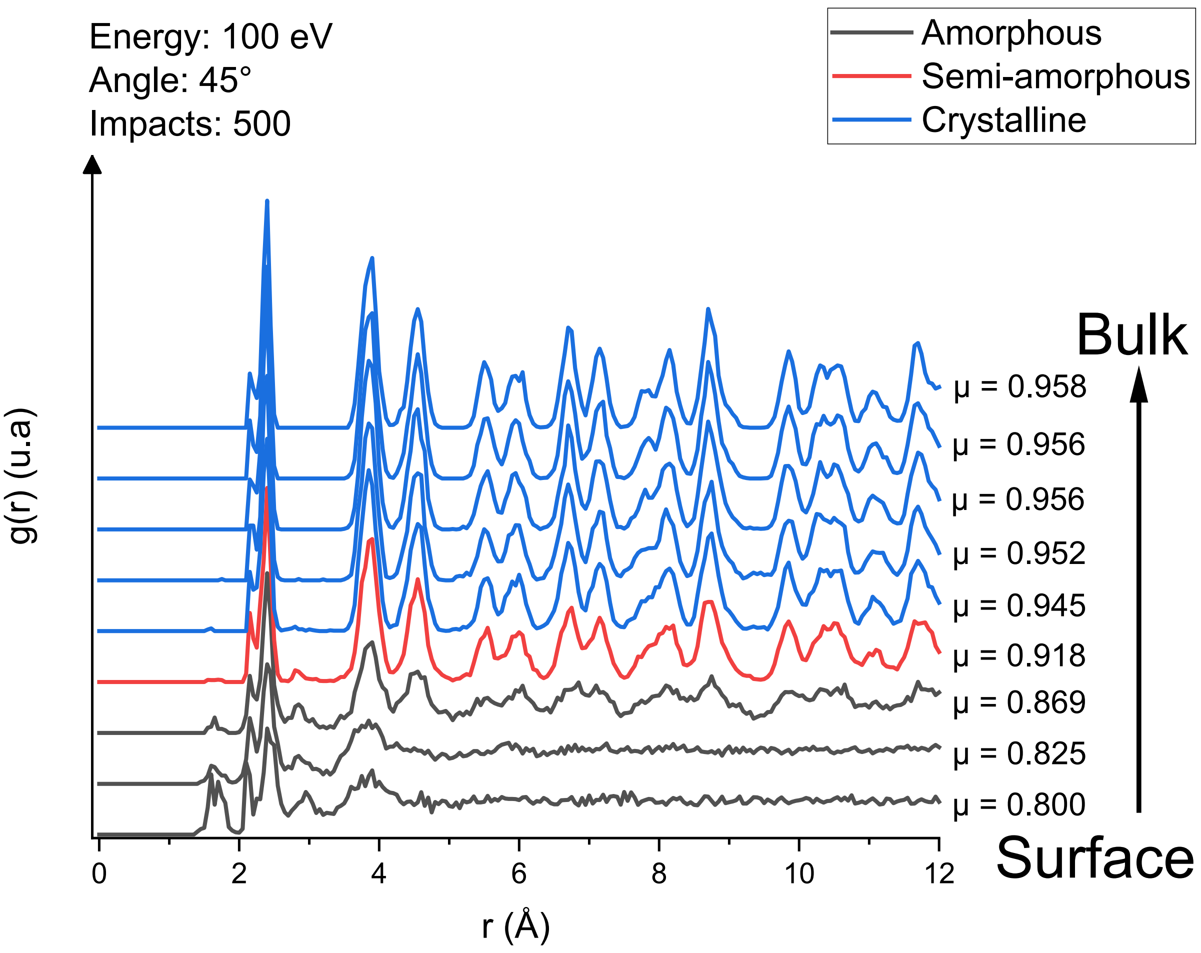

Figure S16. Detailed g(r) for each slab for 100 eV, 75°, and 500 bombardments.

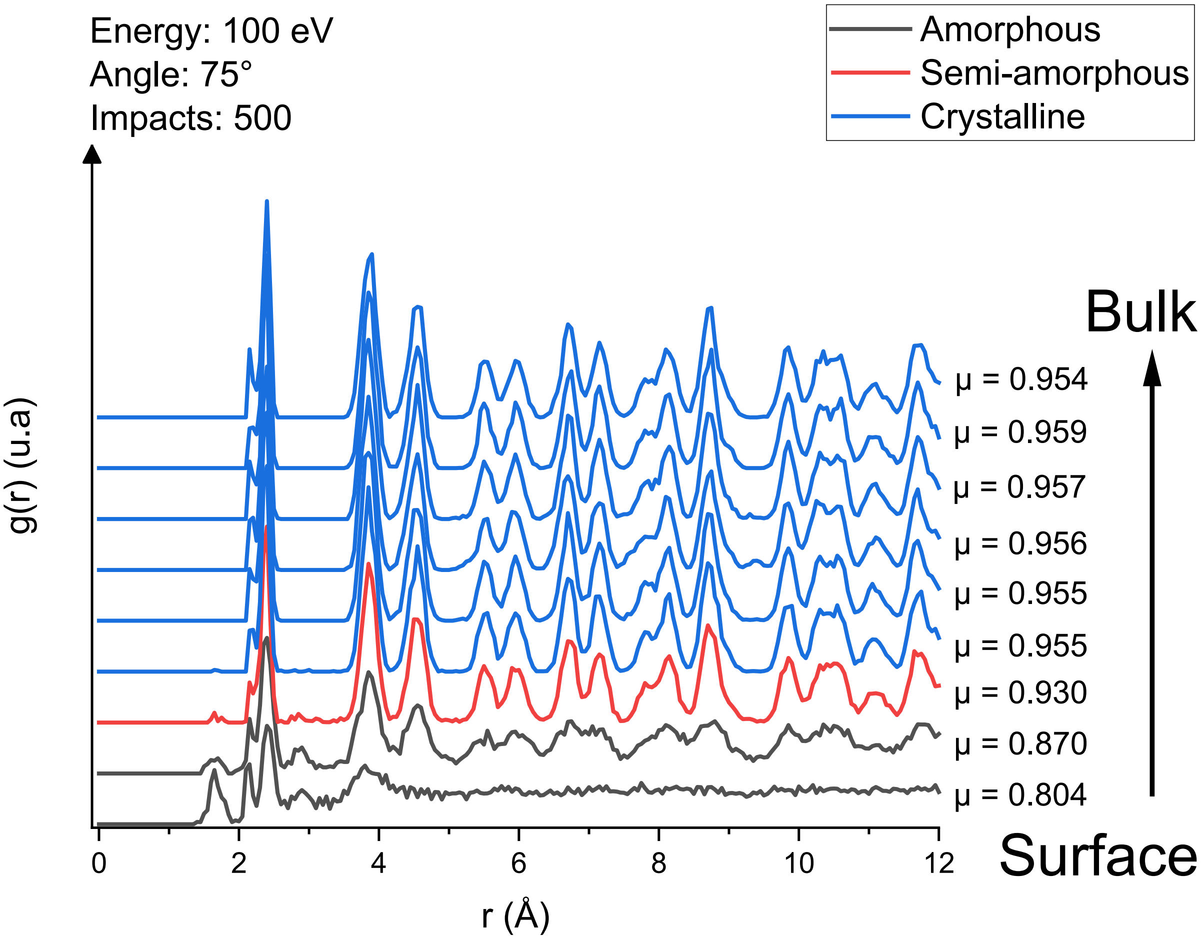

Reaction products

Figure S17. Structure of the raw data for Si–H and Si–O counts at 100 eV and 0° angle of impact.

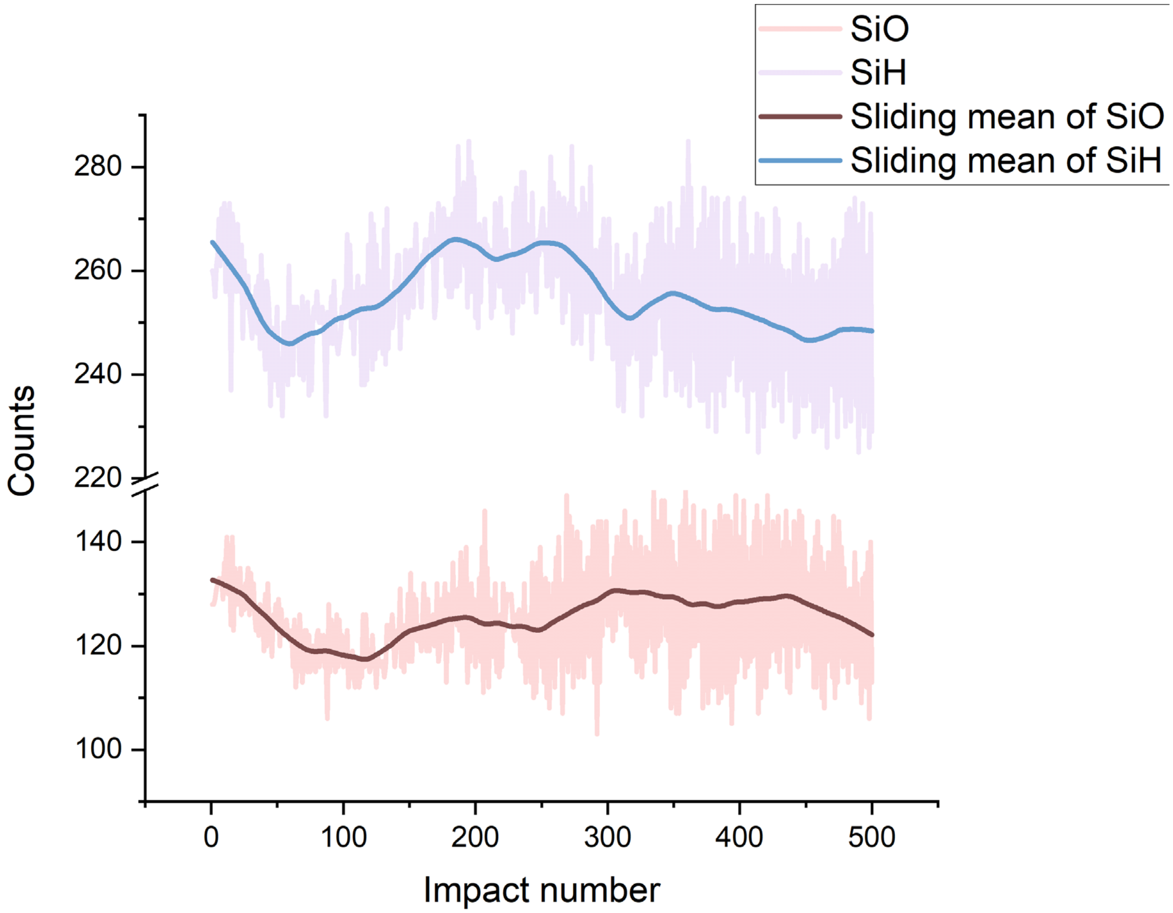

Figure S18. Evolution of the Si–OH products with respect to the fluence.

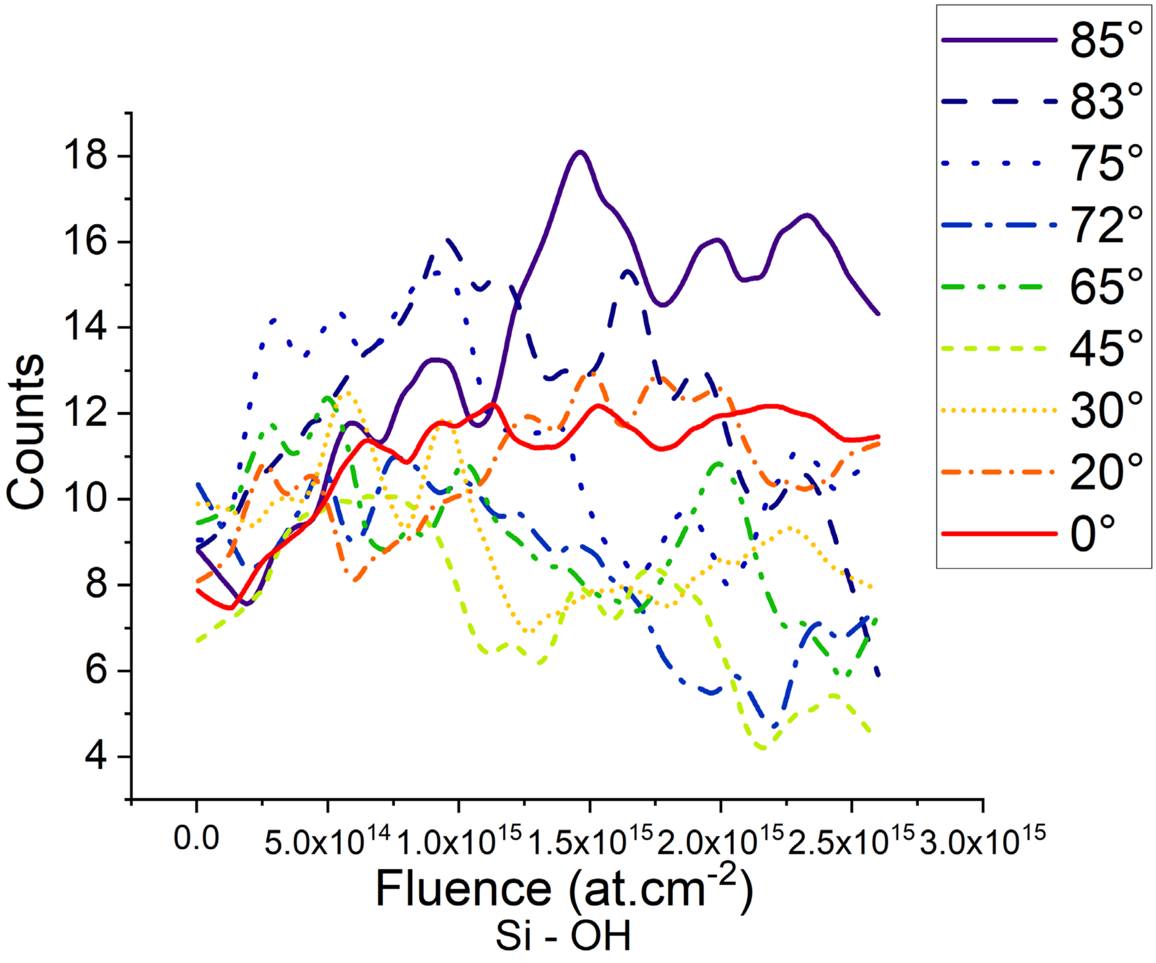

Figure S19. Evolution of the Si–O_2 products with respect to the fluence.

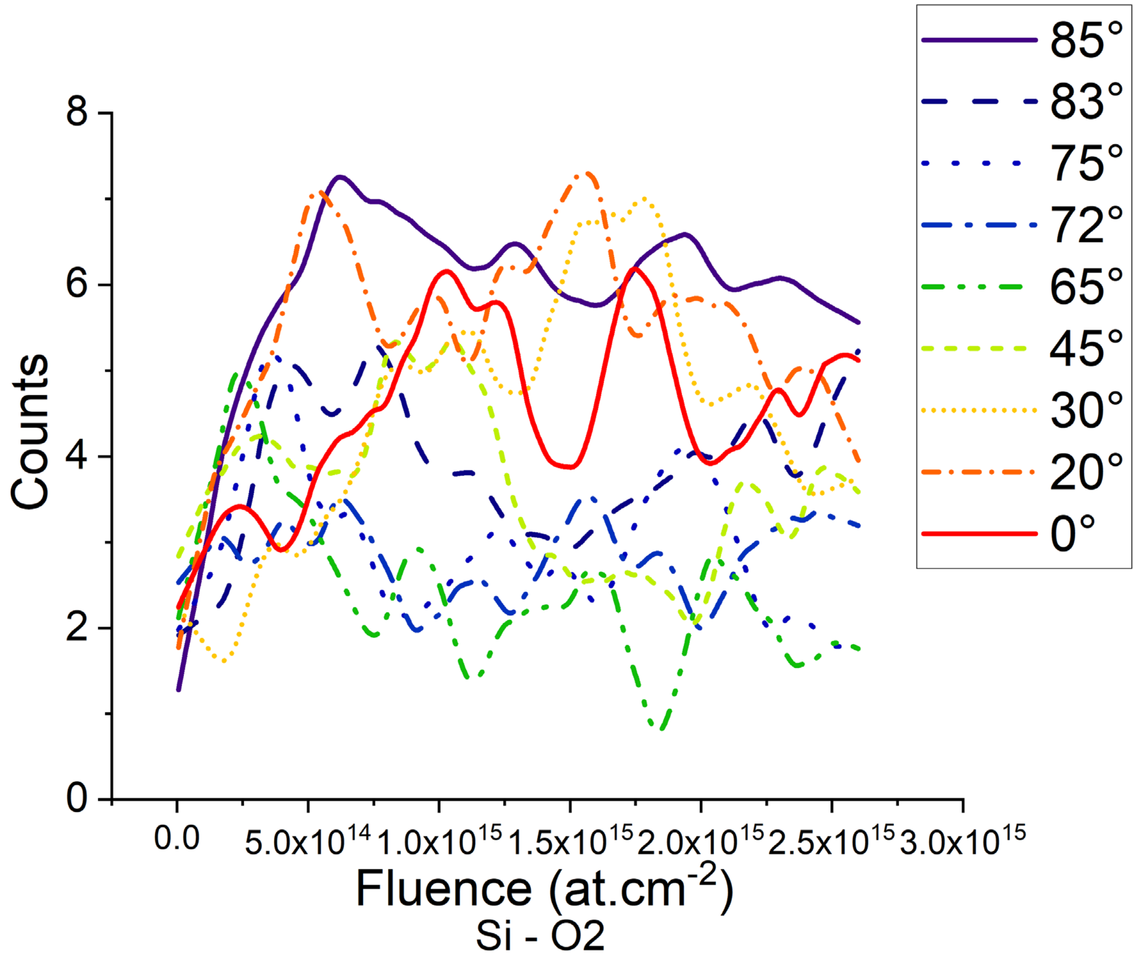

Figure S20. Evolution of the Si–H_2 products with respect to the fluence.

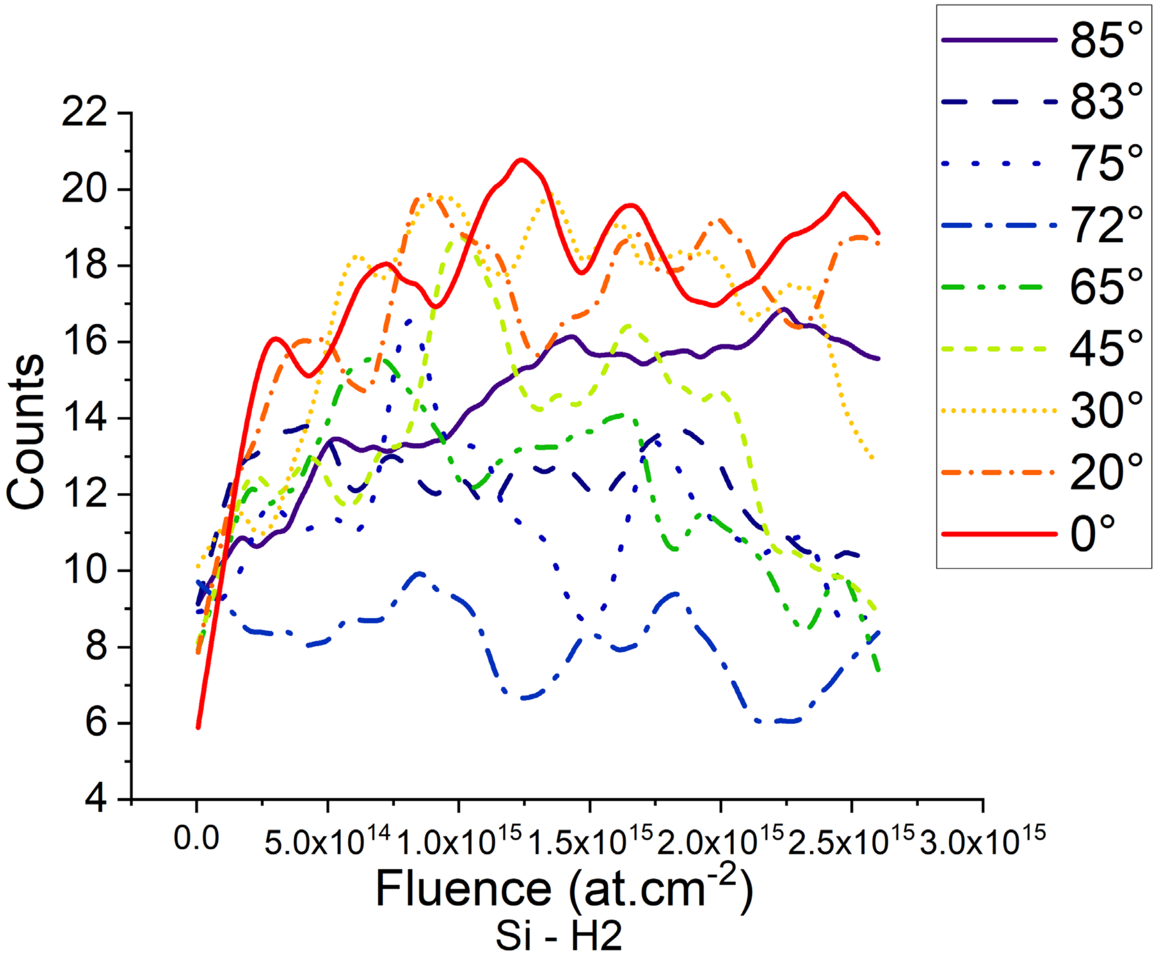

ReaxFF potential

Table S1. Details of the ReaxFF potential used in the simulations.

Reactive MD-force field: c/h/o/Si/C August 3

39 ! Number of general parameters

50.0000 !Overcoordination parameter

9.5469 !Overcoordination parameter

26.5405 !Valency angle conjugation parameter

1.5105 !Triple bond stabilisation parameter

6.6630 !Triple bond stabilisation parameter

0.0000 !C2-correction

1.0588 !Undercoordination parameter

4.6000 !Triple bond stabilisation parameter

12.1176 !Undercoordination parameter

13.3056 !Undercoordination parameter

-70.1292 !Triple bond stabilization energy

0.0000 !Lower Taper-radius

10.0000 !Upper Taper-radius

2.8793 !Not used

33.8667 !Valency undercoordination

6.0891 !Valency angle/lone pair parameter

1.0563 !Valency angle

2.0384 !Valency angle parameter

6.1431 !Not used

6.9290 !Double bond/angle parameter

0.3989 !Double bond/angle parameter: overcoord

3.9954 !Double bond/angle parameter: overcoord

-2.4837 !Not used

5.7796 !Torsion/BO parameter

10.0000 !Torsion overcoordination

1.9487 !Torsion overcoordination

-1.2327 !Conjugation 0 (not used)

2.1645 !Conjugation

1.5591 !vdWaals shielding

0.1000 !Cutoff for bond order (*100)

2.1365 !Valency angle conjugation parameter

0.6991 !Overcoordination parameter

50.0000 !Overcoordination parameter

1.8512 !Valency/lone pair parameter

0.5000 !Not used

20.0000 !Not used

5.0000 !Molecular energy (not used)

0.0000 !Molecular energy (not used)

2.6962 !Valency angle conjugation parameter

7 ! Nr of atoms; cov.r; valency;a.m;Rvdw;Evdw;gammaEEM;cov.r2;#

alfa;gammavdW;valency;Eunder;Eover;chiEEM;etaEEM;n.u.

cov r3;Elp;Heat inc.;n.u.;n.u.;n.u.;n.u.

ov/un;val1;n.u.;val3,vval4

C 1.3825 4.0000 12.0000 1.9133 0.1853 0.9000 1.1359 4.0000

9.7602 2.1346 4.0000 33.2433 79.5548 5.8678 7.0000 0.0000

1.2104 0.0000 199.0303 8.6991 34.7289 13.3894 0.8563 0.0000

-2.8983 2.5000 1.0564 4.0000 2.9663 0.0000 0.0000 0.0000

H 0.7853 1.0000 1.0080 1.5904 0.0419 1.0206 -0.1000 1.0000

9.3557 5.0518 1.0000 0.0000 121.1250 5.3200 7.4366 1.0000

-0.1000 0.0000 62.4879 1.9771 3.3517 0.7571 1.0698 0.0000

-15.7683 2.1488 1.0338 1.0000 2.8793 0.0000 0.0000 0.0000

O 1.2477 2.0000 15.9990 1.9236 0.0904 1.0503 1.0863 6.0000

10.2127 7.7719 4.0000 36.9573 116.0768 8.5000 8.9989 2.0000

0.9088 1.0003 60.8726 20.4140 3.3754 0.2702 0.9745 0.0000

-3.6141 2.7025 1.0493 4.0000 2.9225 0.0000 0.0000 0.0000

N 1.2333 3.0000 14.0000 1.9324 0.1376 0.8596 1.1748 5.0000

10.0667 7.8431 4.0000 32.2482 100.0000 6.8418 6.3404 2.0000

1.0433 13.7673 119.9837 2.1961 3.0696 2.7683 0.9745 0.0000

-4.3875 2.6192 1.0183 4.0000 2.8793 0.0000 0.0000 0.0000

S 1.9401 2.0000 32.0600 2.0629 0.2095 1.0316 1.5483 6.0000

9.9553 4.9055 4.0000 52.9998 112.1416 6.5181 8.2345 2.0000

1.4601 9.6977 71.1843 5.7487 23.2859 12.7147 0.9745 0.0000

-11.0200 2.7266 1.0338 6.2998 2.8793 0.0000 0.0000 0.0000

Si 2.0291 4.0000 28.0600 2.0043 0.1247 0.8218 1.5023 4.0000

13.0000 2.0618 4.0000 11.8211 136.4845 1.8038 7.3852 0.0000

-1.0000 0.0000 126.5182 3.6038 8.5961 0.2368 0.8563 0.0000

-3.5163 4.2105 1.0338 6.2998 2.5791 0.0000 0.0000 0.0000

X -0.1000 2.0000 1.0080 2.0000 0.0000 1.0000 -0.1000 6.0000

10.0000 2.5000 4.0000 0.0000 0.0000 8.5000 15.0000 0.0000

-0.1000 0.0000 127.6226 8.7410 13.3640 0.6690 0.9745 0.0000

-11.0000 2.7466 1.0338 6.2998 2.8793 0.0000 0.0000 0.0000

19 ! Nr of bonds; Edis1;LPpen;n.u.;pbe1;pbo5;13corr;pbo6

pbe2;pbo3;pbo4;n.u.;pbo1;pbo2;ovcorr

1 1 156.5953 100.0397 80.0000 -0.8157 -0.4591 1.0000 37.7369 0.4235

0.4527 -0.1000 9.2605 1.0000 -0.0750 6.8316 1.0000 0.0000

1 2 170.2316 0.0000 0.0000 -0.5931 0.0000 1.0000 6.0000 0.7140

5.2267 1.0000 0.0000 1.0000 -0.0500 6.8315 0.0000 0.0000

2 2 156.0973 0.0000 0.0000 -0.1377 0.0000 1.0000 6.0000 0.8240

2.9907 1.0000 0.0000 1.0000 -0.0593 4.8358 0.0000 0.0000

1 3 160.4802 105.1693 23.3059 -0.3873 -0.1613 1.0000 10.8851 1.0000

0.5341 -0.3174 7.0303 1.0000 -0.1463 5.2913 0.0000 0.0000

3 3 60.1463 176.6202 51.1430 -0.2802 -0.1244 1.0000 29.6439 0.9114

0.2441 -0.1239 7.6487 1.0000 -0.1302 6.2919 1.0000 0.0000

1 4 134.1215 140.2179 79.9745 0.0163 -0.1428 1.0000 27.0617 0.2000

0.1387 -0.3681 7.1611 1.0000 -0.1000 5.0825 1.0000 0.0000

3 4 130.8596 169.4551 40.0000 0.3837 -0.1639 1.0000 35.0000 0.2000

1.0000 -0.3579 7.0004 1.0000 -0.1193 6.8773 1.0000 0.0000

4 4 157.9384 82.5526 152.5336 0.4010 -0.1034 1.0000 12.4261 0.5828

0.1578 -0.1509 11.9186 1.0000 -0.0861 5.4271 1.0000 0.0000

2 3 180.4373 0.0000 0.0000 -0.8074 0.0000 1.0000 6.0000 0.5514

1.2490 1.0000 0.0000 1.0000 -0.0657 5.0451 0.0000 0.0000

2 4 231.8173 0.0000 0.0000 -0.3364 0.0000 1.0000 6.0000 0.4402

8.8910 1.0000 0.0000 1.0000 -0.0327 6.5754 0.0000 0.0000

1 5 129.1942 74.3656 55.2528 0.1066 -0.5211 1.0000 18.9617 0.5950

0.2950 -0.2398 8.0314 1.0000 -0.1019 5.6754 1.0000 0.0000

2 5 151.3159 0.0000 0.0000 -0.4644 0.0000 1.0000 6.0000 0.5950

9.4365 1.0000 0.0000 1.0000 -0.0303 7.0100 1.0000 0.0000

3 5 0.0000 0.0000 0.0000 0.5563 -0.4038 1.0000 49.5611 0.6000

0.4259 -0.4577 12.7569 1.0000 -0.1100 7.1145 1.0000 0.0000

4 5 0.0000 0.0000 0.0000 0.4438 -0.2034 1.0000 40.3399 0.6000

0.3296 -0.3153 9.1227 1.0000 -0.1805 5.6864 1.0000 0.0000

5 5 96.1871 93.7006 68.6860 0.0955 -0.4781 1.0000 17.8574 0.6000

0.2723 -0.2373 9.7875 1.0000 -0.0950 6.4757 1.0000 0.0000

1 6 90.6281 6.3660 0.0000 0.3176 -0.5558 1.0000 17.2117 0.5577

0.7223 -0.2118 7.7440 1.0000 -0.1039 5.4442 1.0000 0.0000

2 6 137.1002 0.0000 0.0000 -0.1902 0.0000 1.0000 6.0000 0.4256

17.7186 1.0000 0.0000 1.0000 -0.0377 6.4281 0.0000 0.0000

3 6 230.7615 93.6959 43.3991 -0.3617 -0.3000 1.0000 36.0000 0.3161

0.9856 -0.3882 4.6686 1.0000 -0.3960 4.5499 1.0000 0.0000

6 6 72.8867 50.0318 30.0000 0.9983 -0.3000 1.0000 16.0000 0.1000

1.0538 -0.0447 10.6176 1.0000 -0.1452 8.0404 0.0000 0.0000

11 ! Nr of off-diagonal terms; Ediss;Ro;gamma;rsigma;rpi;rpi2

1 2 0.1219 1.4000 9.8442 1.1203 -1.0000 -1.0000

2 3 0.0344 1.6800 10.3247 0.9013 -1.0000 -1.0000

2 4 0.1059 1.8290 9.7818 0.9598 -1.0000 -1.0000

1 3 0.1131 1.8523 9.8442 1.2775 1.1342 1.0621

1 4 0.1447 1.8766 9.7990 1.3436 1.1885 1.1363

3 4 0.1048 2.0003 10.1220 1.3173 1.1096 1.0206

1 5 0.1997 2.0109 9.8603 1.6611 1.3423 -1.0000

2 5 0.0938 1.8133 9.6519 1.3629 -1.0000 -1.0000

1 6 0.0250 1.7695 12.4753 1.5866 1.4409 -1.0000

2 6 0.0291 1.6805 12.5137 1.3429 -1.0000 -1.0000

3 6 0.1958 1.7958 11.1207 1.6105 1.1632 -1.0000

71 ! Nr of angles;at1;at2;at3;Thetao,o;ka;kb;pv1;pv2

1 1 1 67.2326 22.0695 1.6286 0.0000 1.7959 15.4141 1.8089

1 1 2 65.2527 14.3185 6.2977 0.0000 0.5645 0.0000 1.1530

2 1 2 70.0840 25.3540 3.4508 0.0000 0.0050 0.0000 3.0000

1 2 2 0.0000 0.0000 6.0000 0.0000 0.0000 0.0000 1.0400

1 2 1 0.0000 3.4110 7.7350 0.0000 0.0000 0.0000 1.0400

2 2 2 0.0000 27.9213 5.8635 0.0000 0.0000 0.0000 1.0400

1 1 3 49.5561 7.3771 4.9568 0.0000 0.7533 15.9906 1.0010

3 1 3 77.1171 39.8746 2.5403 -24.3902 1.7740 -42.9758 2.1240

1 1 4 66.1305 12.4661 7.0000 0.0000 3.0000 50.0000 1.1880

3 1 4 73.9544 12.4661 7.0000 0.0000 3.0000 0.0000 1.1880

4 1 4 64.1581 12.4661 7.0000 0.0000 3.0000 0.0000 1.1880

2 1 3 65.0000 14.2057 4.8649 0.0000 0.3504 0.0000 1.7185

2 1 4 74.2929 31.0883 2.6184 0.0000 0.0755 0.0000 1.0500

1 2 4 0.0000 0.0019 6.3000 0.0000 0.0000 0.0000 1.0400

1 3 1 74.3994 44.7500 0.7982 0.0000 3.0000 0.0000 1.0528

1 3 3 77.9854 36.6201 2.0201 0.0000 0.7434 67.0264 3.0000

1 3 4 82.4890 31.4554 0.9953 0.0000 1.6310 0.0000 1.0783

3 3 3 80.7324 30.4554 0.9953 0.0000 1.6310 50.0000 1.0783

3 3 4 84.3637 31.4554 0.9953 0.0000 1.6310 0.0000 1.0783

4 3 4 89.7071 31.4554 0.9953 0.0000 1.6310 0.0000 1.1519

1 3 2 71.5018 21.7062 0.4735 0.0000 0.5186 0.0000 1.1793

2 3 3 84.9468 23.3540 1.5057 0.0000 2.6374 0.0000 1.3023

2 3 4 75.6201 18.7919 0.9833 0.0000 0.1218 0.0000 1.0500

2 3 2 77.0645 10.4737 1.2895 0.0000 0.9924 0.0000 1.1043

1 4 1 66.0330 22.0295 1.4442 0.0000 1.6777 0.0000 1.0500

1 4 3 103.3204 33.0381 0.5787 0.0000 1.6777 0.0000 1.0500

1 4 4 104.1335 8.6043 1.6495 0.0000 1.6777 0.0000 1.0500

3 4 3 74.1978 42.1786 1.7845 -18.0069 1.6777 0.0000 1.0500

3 4 4 74.8600 43.7354 1.1572 -0.9193 1.6777 0.0000 1.0500

4 4 4 75.0538 14.8267 5.2794 0.0000 1.6777 0.0000 1.0500

1 4 2 69.1106 25.5067 1.1003 0.0000 0.0222 0.0000 1.0369

2 4 3 81.3686 40.0712 2.2396 0.0000 0.0222 0.0000 1.0369

2 4 4 83.0104 43.4766 1.5328 0.0000 0.0222 0.0000 1.0500

2 4 2 70.8687 12.0168 5.0132 0.0000 0.0222 0.0000 1.1243

1 2 3 0.0000 25.0000 3.0000 0.0000 1.0000 0.0000 1.0400

1 2 4 0.0000 0.0019 6.0000 0.0000 0.0000 0.0000 1.0400

1 2 5 0.0000 0.0019 6.0000 0.0000 0.0000 0.0000 1.0400

3 2 3 0.0000 0.0148 6.0000 0.0000 0.0000 0.0000 1.0400

3 2 4 0.0000 0.0019 6.0000 0.0000 0.0000 0.0000 1.0400

4 2 4 0.0000 0.0019 6.0000 0.0000 0.0000 0.0000 1.0400

2 2 3 0.0000 9.7025 6.0000 0.0000 0.0000 0.0000 1.0400

2 2 4 0.0000 0.0019 6.0000 0.0000 0.0000 0.0000 1.0400

1 1 5 73.9923 24.7559 1.8287 0.1463 0.0059 0.0000 1.0600

1 5 1 86.7521 36.5756 2.0199 0.1463 0.0058 0.0000 1.0600

2 1 5 75.1310 24.8619 1.8104 0.0000 0.0050 0.0000 1.0600

1 5 2 85.3326 36.9451 2.1403 0.0000 0.0388 0.0000 1.0706

1 5 5 86.0081 37.0451 2.1403 0.1463 0.1070 0.0000 1.0098

2 5 2 92.9959 36.9602 2.0403 0.0000 0.0050 0.0000 1.0200

2 5 5 83.2918 36.9451 2.0199 0.0000 0.0050 0.0000 1.0600

2 2 5 0.0000 0.0019 6.0000 0.0000 0.0000 0.0000 1.0400

6 6 6 71.6771 13.0081 3.6376 0.0000 0.2384 0.0000 1.3185

2 6 6 89.1207 11.7566 1.1579 0.0000 0.0100 0.0000 1.2975

2 6 2 26.3763 5.5393 0.9656 0.0000 2.3381 0.0000 1.1704

3 6 6 85.6335 17.1826 6.5759 0.0000 0.4105 0.0000 1.6398

2 6 3 59.6558 6.8748 7.0452 0.0000 4.0000 0.0000 1.0400

3 6 3 72.7359 17.5203 2.4434 0.0000 0.0100 0.0000 1.7374

6 3 6 18.3653 5.7702 3.4915 0.0000 4.0000 0.0000 1.9438

2 3 6 57.5894 40.0000 8.0000 0.0000 3.8263 0.0000 1.0534

3 3 6 54.5893 38.8349 7.6245 0.0000 2.7656 0.0000 3.0000

2 2 6 0.0000 47.1300 6.0000 0.0000 1.6371 0.0000 1.0400

6 2 6 0.0000 31.5209 6.0000 0.0000 1.6371 0.0000 1.0400

3 2 6 0.0000 31.0427 4.5625 0.0000 1.6371 0.0000 1.0400

1 1 6 63.8858 35.1811 0.6236 0.0000 2.6344 0.0000 2.3890

1 6 1 71.6429 31.1160 0.5107 0.0000 0.0100 0.0000 1.9113

6 1 6 63.2523 33.3810 2.2952 0.0000 0.0201 0.0000 1.7191

1 6 6 70.9876 29.7098 1.0210 0.0000 0.0100 0.0000 1.8242

2 1 6 96.9319 10.9008 1.4627 0.0000 2.4557 0.0000 1.5109

1 6 2 73.9320 16.6559 3.0433 0.0000 0.7961 0.0000 1.4005

1 3 6 91.5678 5.9243 2.4284 0.0000 2.9840 0.0000 1.0400

1 6 3 96.3796 36.5757 0.8505 0.0000 3.6964 0.0000 1.6527

3 1 6 42.5553 40.0000 1.5855 0.0000 1.0802 0.0000 1.1584

34 ! Nr of torsions;at1;at2;at3;at4;;V1;V2;V3;V2(BO);vconj;n.u;n

1 1 1 1 -0.2500 11.5822 0.1879 -4.7057 -2.2047 0.0000 0.0000

1 1 1 2 -0.2500 31.2596 0.1709 -4.6391 -1.9002 0.0000 0.0000

2 1 1 2 -0.1770 30.0252 0.4340 -5.0019 -2.0697 0.0000 0.0000

1 1 1 3 -0.7098 22.2951 0.0060 -2.5000 -2.1688 0.0000 0.0000

2 1 1 3 -0.3568 22.6472 0.6045 -4.0088 -1.0000 0.0000 0.0000

3 1 1 3 -0.0528 6.8150 0.7498 -5.0913 -1.0000 0.0000 0.0000

1 1 3 1 2.0007 25.5641 -0.0608 -2.6456 -1.1766 0.0000 0.0000

1 1 3 2 -1.1953 42.1545 -1.0000 -8.0821 -1.0000 0.0000 0.0000

2 1 3 1 -0.9284 34.3952 0.7285 -2.5440 -2.4641 0.0000 0.0000

2 1 3 2 -2.5000 79.6980 1.0000 -3.5697 -2.7501 0.0000 0.0000

1 1 3 3 -0.0179 5.0603 -0.1894 -2.5000 -2.0399 0.0000 0.0000

2 1 3 3 -0.5583 80.0000 1.0000 -4.4000 -3.0000 0.0000 0.0000

3 1 3 1 -2.5000 76.0427 -0.0141 -3.7586 -2.9000 0.0000 0.0000

3 1 3 2 0.0345 78.9586 -0.6810 -4.1777 -3.0000 0.0000 0.0000

3 1 3 3 -2.5000 66.3525 0.3986 -3.0293 -3.0000 0.0000 0.0000

1 3 3 1 2.5000 -0.5332 1.0000 -3.5096 -2.9000 0.0000 0.0000

1 3 3 2 -2.5000 3.3219 0.7180 -5.2021 -2.9330 0.0000 0.0000

2 3 3 2 2.2500 -6.2288 1.0000 -2.6189 -1.0000 0.0000 0.0000

1 3 3 3 0.0531 -17.3983 1.0000 -2.5000 -2.1584 0.0000 0.0000

2 3 3 3 0.4723 -12.4144 -1.0000 -2.5000 -1.0000 0.0000 0.0000

3 3 3 3 -2.5000 -25.0000 1.0000 -2.5000 -1.0000 0.0000 0.0000

0 1 2 0 0.0000 0.0000 0.0000 0.0000 0.0000 0.0000 0.0000

0 2 2 0 0.0000 0.0000 0.0000 0.0000 0.0000 0.0000 0.0000

0 2 3 0 0.0000 0.1000 0.0200 -2.5415 0.0000 0.0000 0.0000

0 1 1 0 0.0000 50.0000 0.3000 -4.0000 -2.0000 0.0000 0.0000

0 3 3 0 0.5511 25.4150 1.1330 -5.1903 -1.0000 0.0000 0.0000

0 1 4 0 -2.4242 128.1636 0.3739 -6.6098 -2.0000 0.0000 0.0000

0 2 4 0 0.0000 0.1000 0.0200 -2.5415 0.0000 0.0000 0.0000

0 3 4 0 1.4816 55.6641 0.0004 -7.0465 -2.7831 0.0000 0.0000

0 4 4 0 -0.3244 27.7086 0.0039 -2.8272 -2.0000 0.0000 0.0000

4 1 4 4 -5.5181 8.9706 0.0004 -6.1782 -2.0000 0.0000 0.0000

0 1 5 0 0.1515 29.0501 0.0792 -4.5064 -1.0200 0.0000 0.0000

0 5 5 0 -0.0054 0.1000 0.1715 -2.2256 -1.0000 0.0000 0.0000

0 2 5 0 0.0000 0.0000 0.0000 0.0000 0.0000 0.0000 0.0000

0 ! Nr of hydrogen bonds;at1;at2;at3;Rhb;Dehb;vhb1

3 2 3 1.9682 -4.4628 1.7976 3.0000

3 2 4 2.0000 -6.0000 1.7976 3.0000

4 2 3 1.2000 -2.0000 1.7976 3.0000

4 2 4 1.2979 -6.0000 1.7976 3.0000

3 2 5 1.5000 -2.0000 1.7976 3.0000

4 2 5 1.5000 -2.0000 1.7976 3.0000

5 2 3 1.5000 -2.0000 1.7976 3.0000

5 2 4 1.5000 -2.0000 1.7976 3.0000

5 2 5 1.5000 -2.0000 1.7976 3.0000
